# Magnetic Nanoparticles as Mediators of Ligand-Free Activation of EGFR Signaling

**DOI:** 10.1371/journal.pone.0068879

**Published:** 2013-07-23

**Authors:** Atul A. Bharde, Raghavendra Palankar, Cornelia Fritsch, Arjen Klaver, Johannes S. Kanger, Thomas M. Jovin, Donna J. Arndt-Jovin

**Affiliations:** 1 Laboratory of Cellular Dynamics, Max Planck Institute for Biophysical Chemistry, Göttingen, Germany; 2 Nanobiophysics, Faculty of Science and Technology, University of Twente, Enschede, The Netherlands; Brandeis University, United States of America

## Abstract

**Background:**

Magnetic nanoparticles (NPs) are of particular interest in biomedical research, and have been exploited for molecular separation, gene/drug delivery, magnetic resonance imaging, and hyperthermic cancer therapy. In the case of cultured cells, magnetic manipulation of NPs provides the means for studying processes induced by mechanotransduction or by local clustering of targeted macromolecules, e.g. cell surface receptors. The latter are normally activated by binding of their natural ligands mediating key signaling pathways such as those associated with the epidermal growth factor (EGFR). However, it has been reported that EGFR may be dimerized and activated even in the absence of ligands. The present study assessed whether receptor clustering induced by physical means alone suffices for activating EGFR in quiescent cells.

**Methodology/Principal Findings:**

The EGFR on A431 cells was specifically targeted by superparamagnetic iron oxide NPs (SPIONs) carrying either a ligand-blocking monoclonal anti-EGFR antibody or a streptavidin molecule for targeting a chimeric EGFR incorporating a biotinylated amino-terminal acyl carrier peptide moiety. Application of a magnetic field led to SPION magnetization and clustering, resulting in activation of the EGFR, a process manifested by auto and transphosphorylation and downstream signaling. The magnetically-induced early signaling events were similar to those inherent to the ligand dependent EGFR pathways. Magnetization studies indicated that the NPs exerted magnetic dipolar forces in the sub-piconewton range with clustering dependent on Brownian motion of the receptor-SPION complex and magnetic field strength.

**Conclusions/Significance:**

We demonstrate that EGFR on the cell surface that have their ligand binding-pocket blocked by an antibody are still capable of transphosphorylation and initiation of signaling cascades if they are clustered by SPIONs either attached locally or targeted to another site of the receptor ectodomain. The results suggest that activation of growth factor receptors may be triggered by ligand-independent molecular crowding resulting from overexpression and/or sequestration in membrane microdomains.

## Introduction

Nanoparticles differing in composition, shape, size, and intrinsic optical, electronic and magnetic properties have been used in diverse biological applications such as imaging, sensing and separation [1], [2], [3], [4]. In particular, magnetic NPs [5] have been exploited for molecular separation, gene/drug delivery, and magnetic resonance imaging [6], [7]. As sensors and actuators they have been used to sense femtomolar concentrations of proteins, mRNA or viruses [8], for focused heat-induced manipulation of ion channels [9], or for mechanotransduction of ion channels in neurons [10]. Some cell surface receptors are activated by clustering, a prominent example being the FcεR1 receptor on basophils and mast cells that is aggregated upon recognition of multivalent allergens by bound IgE [11]. Mannix et al. demonstrated that monovalent antigen attached to SPIONs could induce mast cell activation, manifested by Ca^2+^ waves arising after clustering the FcεR1 by a magnetic field [12]. Apoptosis of tumor cells has been achieved by magnetic aggregation of SPIONs coupled to a monoclonal antibody against DR4 receptors [13], although it was necessary to apply the magnetic field for 2 hr in order to observe caspase 3 activity. The same group achieved a similar result in live zebrafish embryos by targeting the ovarian TNF receptor with microinjected SPIONs and applying a field for 24 or 48 h. A number of recent studies have utilized large magnetic NPs introduced by microinjection to redistribute materials inside cells. Examples are cytoskeletal reorganization induced by Raf1 NPs [14] and microtubule assembly in Xenopus oocyte extracts by RANQ-GTP coupled NPs [15].

The epidermal growth factor receptor (EGFR, ErbB1, HER1), a prototypic transmembrane tyrosine kinase receptor, is a member of the ErbB (HER) family. Ligand binding results in dimerization and subsequent trans-phosphorylation of several tyrosine residues in the intracellular C-terminal tail of the receptor [16], [17], [18]. The adaptor proteins Shc, Grb2 and Cbl recognize these phosphotyrosines, thereby propagating downstream signaling, effector functions and receptor internalization [19], [20]. These signaling cascades orchestrate a wide range of cellular processes such as cell differentiation, motility, and cell division [21], [22].

It has not been firmly established whether receptor dimers or oligomers can be activated and initiate downstream signaling in the absence of physiological ligands. Yu et al. [23] reported that EGFR dimerized and was activated merely by association with α2β1 integrins in serum deprived cells while Takahashi et al. [24] studied the effect of extracellular matrix glycans on ligand free activation of ErbB3 mutants. However, another investigation of integrin association by Alexi et al. [25] failed to demonstrate EGFR activation without added ligand, and the authors concluded that autocrine activation of the receptor was likely to have occurred in some of the other studies. Monoclonal antibodies that block ligand binding inhibit EGFR signaling and some cause down regulation of the receptor [26], [27], [28], suggesting that ligand binding is indeed required for EGFR activation. Some of these antibodies have been humanized and used to treat cancers expressing high levels of EGFR [29], [30], [31]. Certain mutations of EGFR cause a constitutive ligand independent activation of the receptor and such forms often arise under conditions favoring cellular transformation [32], [33].

We initiated the present study to determine whether clustering of EGFR on living cells could bypass the ligand requirement for activating the receptor. We exploited the biocompatibility, tunable surface properties and ease of preparation of superparamagnetic iron oxide NPs (SPIONs), employing the latter as magnetic actuators (“switches”). Non-ligand mediated clustering activated the EGFR and led to internalization of both the receptor and the SPIONs.

## Results

The properties of magnetic nanostructures depend on size. Below a certain critical dimension they exhibit the phenomenon of superparamagnetism, which is characterized by a high magnetic susceptibility and the absence of a residual field [3], [34], [35]. SPIONs were synthesized by alkaline co-precipitation using citrate as a stabilizing agent [34], [35]. Transmission electron microscopy (TEM) images indicated the presence of well-defined, spherical shaped particles 12–15 nm in diameter (Figure S1a–e). A more homogenous SPION population was obtained by magnetic fractionation followed by functionalization with streptavidin (strv-SPION) (see Materials and Methods).

### Magnetic Switches are Created by Covalently Attaching Cell-targeting Molecules to SPION

**Figure 1:**
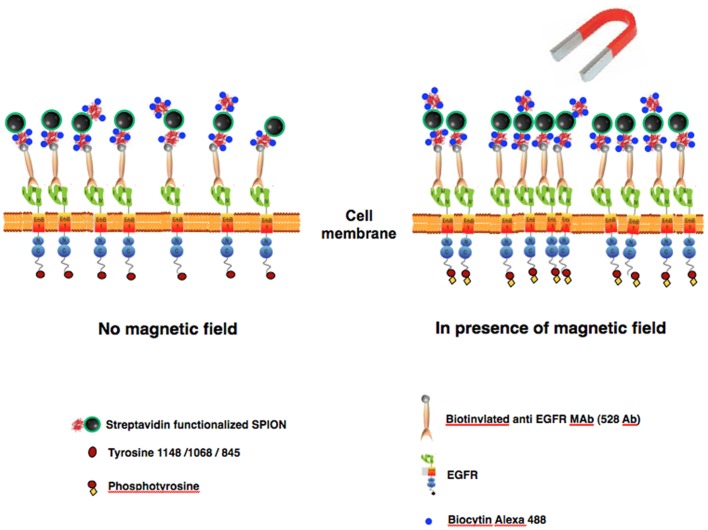
Schematic depiction of the composition and use targeted SPION magnetic switches for ligand-free activation of EGFR in a cell membrane. Magnetic switches (MS) consisting of SPION covalently coupled to streptavidin further reacted with biotinylated anti-EGFR MAb, in most cases, and biocytin-fluorophore.

Figure 1 shows pictorially the composition of “magnetic switches” (MS) and the experimental approach for specific targeting to EGFR. MS were created by coupling strv-SPION to a biotinylated anti-EGFR (ectodomain) monoclonal antibody (528 MAb). This antibody blocks binding of the normal ligands for EGFR, and thus inhibits ligand-induced activation as well as cell proliferation and orthotopic tumor growth [36], [37]. We adjusted the reagent concentrations to achieve ≤1 streptavidin and ∼1 MAb per nanoparticle. We also demonstrated that strv-SPIONs by themselves can act as MS if targeted to cells expressing a chimeric EGFR with a biotinylated acyl carrier protein (ACP) tag on the amino terminus of the receptor (see Materials and Methods). In order to visualize the MS on the cell surface by fluorescence, unoccupied biotin binding sites on strv-SPION were loaded with biocytin-Alexa 488 or biocytin-Alexa 546.

### SPIONS Aggregate and Cause Activation of the Membrane-bound EGFR Only in the Presence of a Magnetic Field

**Figure 2:**
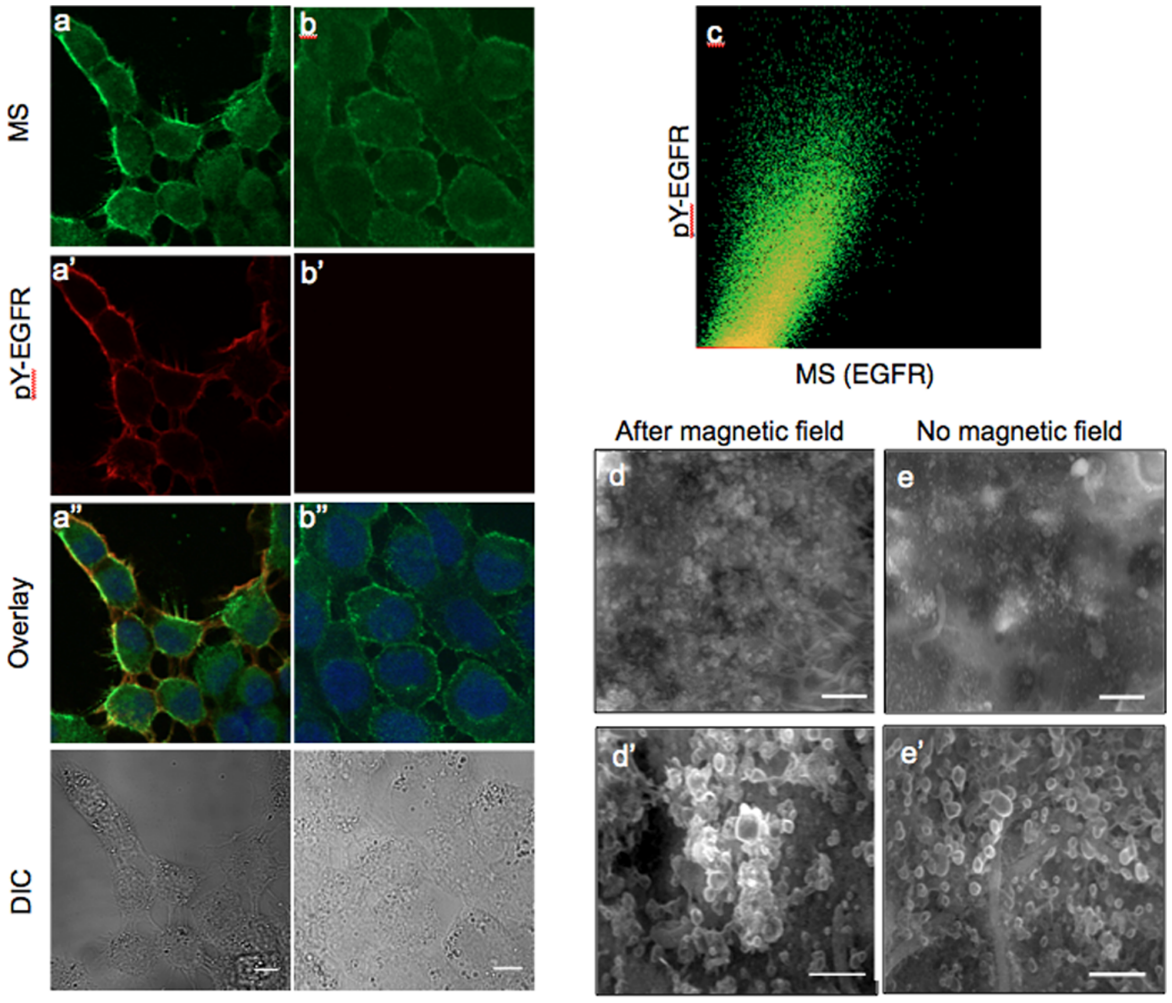
Magnetic field induced activation of EGFR. Confocal image analysis-left column after 60 sec magnetization or right column without magnetiztion. (a,b) MS- Alexa488-biocytin, green; (a′, b′) EGFR activation (pY-EGFR1068) red; (a′′,b′′) overlay of red, green and DAPI DNA staining, blue, images; and DIC image (bottom). Scale bar is 10 µm. (c) Two-dimensional colocalization histogram for the same confocal sections a and a′ after background subtraction. SEM images of A431 cells reacted with MS after (d,d′) and without (e,e′) application of magnetic field. Scale bars, 1 µm for (d & e) and 500 nm for (d′ & e′), respectively.

Application of the targeted MS to A431 cells and washing to remove unbound particles resulted in MS distributed over the entire cell surface (Figure S2a–d). Untargeted SPIONs (lacking anti-EGFR) did not bind to the cells (Figure S2e). Exposure to an external magnetic field led to aggregation of MS into small clusters (Figure 2a) and activation of EGFR detected by an EGFR-specific anti-phosphotyrosine (pY) antibody (Fig. 2a′). In contrast, no EGFR-specific pY signal was observed in cells that were not exposed to a magnetic field after binding of MS (Figure 2b,b′). Figure 2c shows a 2-dimensional colocalization histogram of EGFR phosphorylation and the MS signals from the images in Figure 2a,a′, revealing a strong, positive correlation (colocalization parameters: Manders coefficient 0.78, overlapping coefficient 0.82, and correlation coefficient 0.80).

In order to define the dipolar interactions and the anticipated extent of magnetic field induced clustering of MS bound to EGFR on the cell surface, we carried out a detailed theoretical analysis of the experimental system. Finite element analysis calculations indicated that the magnetic field experienced by MS on the cell surface would be close to saturating strength (Figure S3). At the separation distance of 10 nm the calculated force between neighboring MS on cell surface was ∼ 0.25 pN, decaying exponentially with increasing separation distance. We conclude that in our experimental system the strengths of the magnetic dipole moment and of the external field sufficed for inducing the MS to form stable clusters on the cell surface (Supporting Information S1 and Figure S4).

Scanning electron microscopy (SEM) resolved the local clustering of MS targeted to EGFR on A431 cell membranes. In the absence of a magnetic field, MS were distributed densely on the cell surface but were individually distinct with little or no clustering (Figure 2d,d′). In contrast, cells treated with MS in the presence of a magnetic field showed a dense population of particles arranged in local clusters of ∼ 100–200 nm (Figure 2e,e′).

MS did not exhibit magnetic hysteresis at room temperature, indicating the existence of superparamagnetic behavior (Figure S3). Based on the magnetic properties, we calculated the magnetic diameter of MS to be ∼ 10 nm (see Supporting Information S1), a result which was corroborated by TEM and the estimation of the hydrodynamic diameter as ∼15 nm by dynamic light scattering (Fig. S1).

### EGFR Activation is Dependent on the Time of Magnetic Exposure

**Figure 3:**
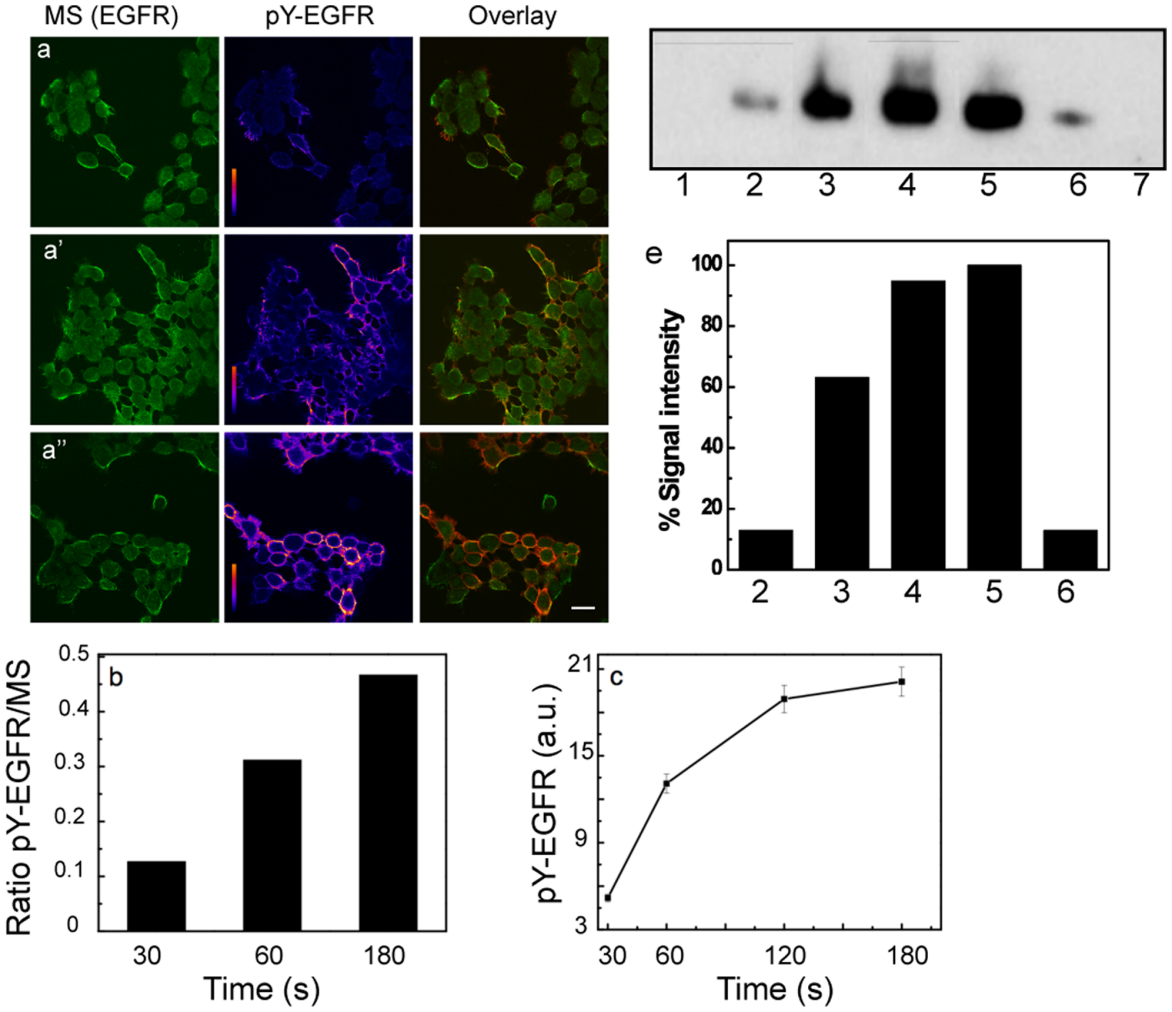
Effect of magnetization time on the level of EGFR phosphorylation. (a, a′, a′′) The time axis shown vertically. Confocal images of MS 488Alexa biocytin signal (green, left panels) and of anti-pY-EGFR 1068 and GARIG-Cy5 (rainbow intensity scale, middle panels) on A431 cells as a function of the applied magnetic field for time intervals of 30, 60 and 180 s. Overlay images are depicted with green/red LUTs, Alexa488-biocytin/GARIG-Cy5 respectively (right panels). Scale bar, 10 µm. (b) Fluorescence intensity ratio of pY-EGFR to MS signals as a function of magnetization. (c) Mean pixel intensity of the pY-EGFR signal from 5 images for each time point as a function of MS magnetization time. (e) Western blot analysis of A431 cell extracts for pY-EGFR 1148. Lane 1, sample obtained from A431 cells incubated with MS in the absence of a magnetic field. Lanes 2–4, pY-EGFR signals for 30, 60 or 180 s of MS magnetization. Lane 5 and 6, pY-EGFR signals after treatment with 30 nM (saturating) or 100 pM EGF. Lane 7, extract of untreated cells. (e) Signal intensities of the positive pY-EGFR lanes in (d) relative to the lane from 30 nM EGF treated cells.

To determine the effect of magnetization as a function of time, MS targeted to EGFR in A431 cells were exposed to a magnetic field for various time periods and the resulting pY-EGFR signals were measured by image analysis (Figure 3a,b,c) and western blotting (Figure 3d,e). Exposures as short as a few seconds showed receptor activation whereas no pY-EGFR was detected in the absence of a magnetic field. Distinct pY-EGFR signals were observed and increased for up to 3 min of magnetization (Figure 3b,c) but then remained constant, indicating that maximum activation had been achieved with the given field. Quantification by western blot analysis (Figure 3 d.e) indicated similar signal intensities for pY-EGFR at saturating concentrations of natural ligand and after a 3 min exposure of the MS to a magnetic field. These observations were consistent with measured magnetization data for the SPIONs (Figures S3 and S4 and Supporting Information S1).

### Magnetic Switches Induce Downstream Signaling of the EGFR

**Figure 4:**
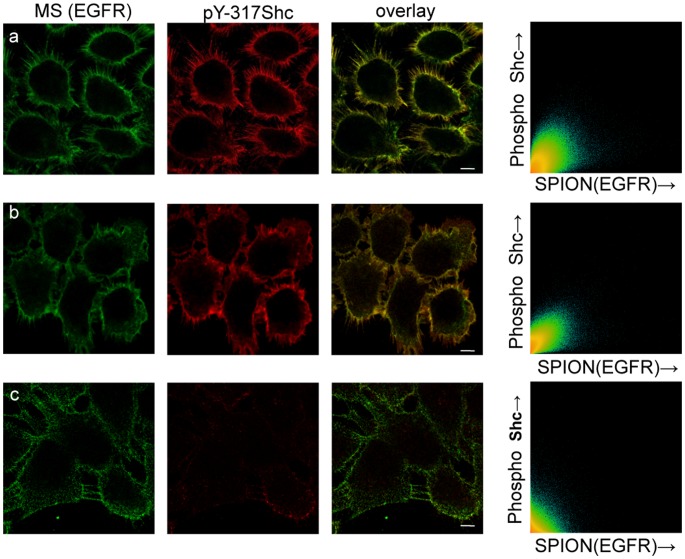
Shc activation as a result of magnetic activation of EGFR. Confocal immunofluorescence images of cells incubated for 15 min at 37°C after 0 sec (a) or after 30 sec (b) or 180 sec (c) magnetic field activation. Image columns left to right: MS Alexa-488 biocytin (green); indirect immunofluorescence of MAb against pY-317 Shc protein and GARIG-CY5 (red); overlay of the first 2 columns; two-dimensional colocalization histograms of MS and pY317-Shc fluorescence signals after deconvolution of 50 optical sections using SVI Huygens software.

**Figure 5:**
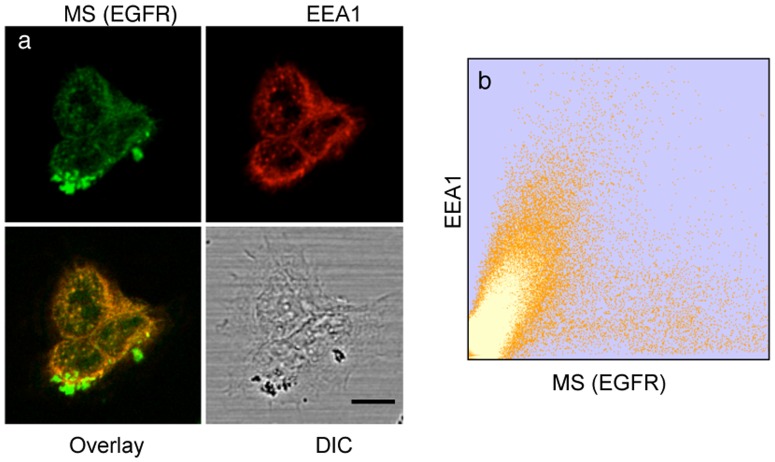
Localization of MS in endocytic vesicles after magnetic field pulse and 37°C incubation. (a) Confocal microscopy images showing the endocytosis of MS after 3 min magnetization and subsequent incubation for 20 min at 37°C in the absence of magnetic field. Green, MS signal; red, immunofluorescence staining of early endosomes by Mab EEA1 and GAMIG-CY3. Scale bar is 10 µm. (b) Two-dimensional colocalization histogram of MS and EEA1 from a z stack (0.7 µm sections) of 20 images.

To determine whether magnetism-induced activation of the EGFR triggers the same signal cascade as that induced by ligand binding, we investigated the recruitment, redistribution and state of phosphorylation of proteins in the MAP Kinase pathway. Shc (Src homology and collagen) is an early adapter protein that is recruited to the plasma membrane after EGF stimulation, where it binds specific phosphotyrosines in the C-terminus of the activated receptor and is itself phosphorylated by the receptor [38], [39]. We induced activation of EGFR by magnetization of tagged MS and assayed by immunofluorescence the appearance as well as the colocalization of p317-Shc with the SPIONs after further incubation of the cells for 10 min at 37 °C, compared with cells not exposed to the magnetic field (Figure 4 a,b,c). The two-dimensional colocalization histograms are shown for each condition and were derived after deconvolution of image stacks using SVI Huygens software. The Manders coefficient was 0.95 after 30 s and 0.98 for 180 s of magnetization, respectively. Subsequent to signaling by EGF at the plasma membrane, the receptor was endocytosed through clathrin-coated pits [40], [41]. We observed this phenomenon after activation by SPIONs, which colocalized with the early endosomal marker EEA1 in cells transduced magnetically for 3 min and then incubated for 15 min at 37°C (Figure 5a and b). In the absence of the magnetic field the MS remained dispersed on the cell surface and there was no activation (phosphorylation) during incubation at 37°C, whereas MS clustering and activation of EGFR was evident after application of the magnetic field for 30 s and subsequent incubation at 37°C (Figure S5 a–d). Simulations for the cluster formation dynamics and Brownian relaxation for such particles are given in the Supporting Information S1 and Figure S6.

### High Order Clustering of the EGFR is Required for Activation by MS

Bivalent crosslinking of biotinylated 528 MAb bound to EGFR mediated by streptavidin-Atto565 (Figure S7) did not result in a detectable pY-EGFR signal, indicating that protein bridging between the MAb bound to the receptor was incapable of associating the EGFR with the kinase domainsoriented in an active conformation. We conclude that higher order clustering of the EGFR and/or a more intimate contact of the receptors are required and can be achieved only by application of the magnetic field.

### Magnetic Activation of EGFR by Targeted SPIONs does not Depend on High Expression Levels of the Receptor on the Cell Membrane or on Targeting by a Large Monoclonal Antibody

To determine whether MS were effective only on cells with very high densities of receptor, we performed the same experiments on Hela cells expressing 5·10^4^ EGFR, i.e. much less than the 2·10^6^ receptors characteristic of A431 cells. Figure S8 shows that EGFR on Hela cells was activated by magnetization of bound MS, demonstrating that activation by local clustering is a phenomenon that can be generalized to other cell types with low receptor densities.

**Figure 6:**
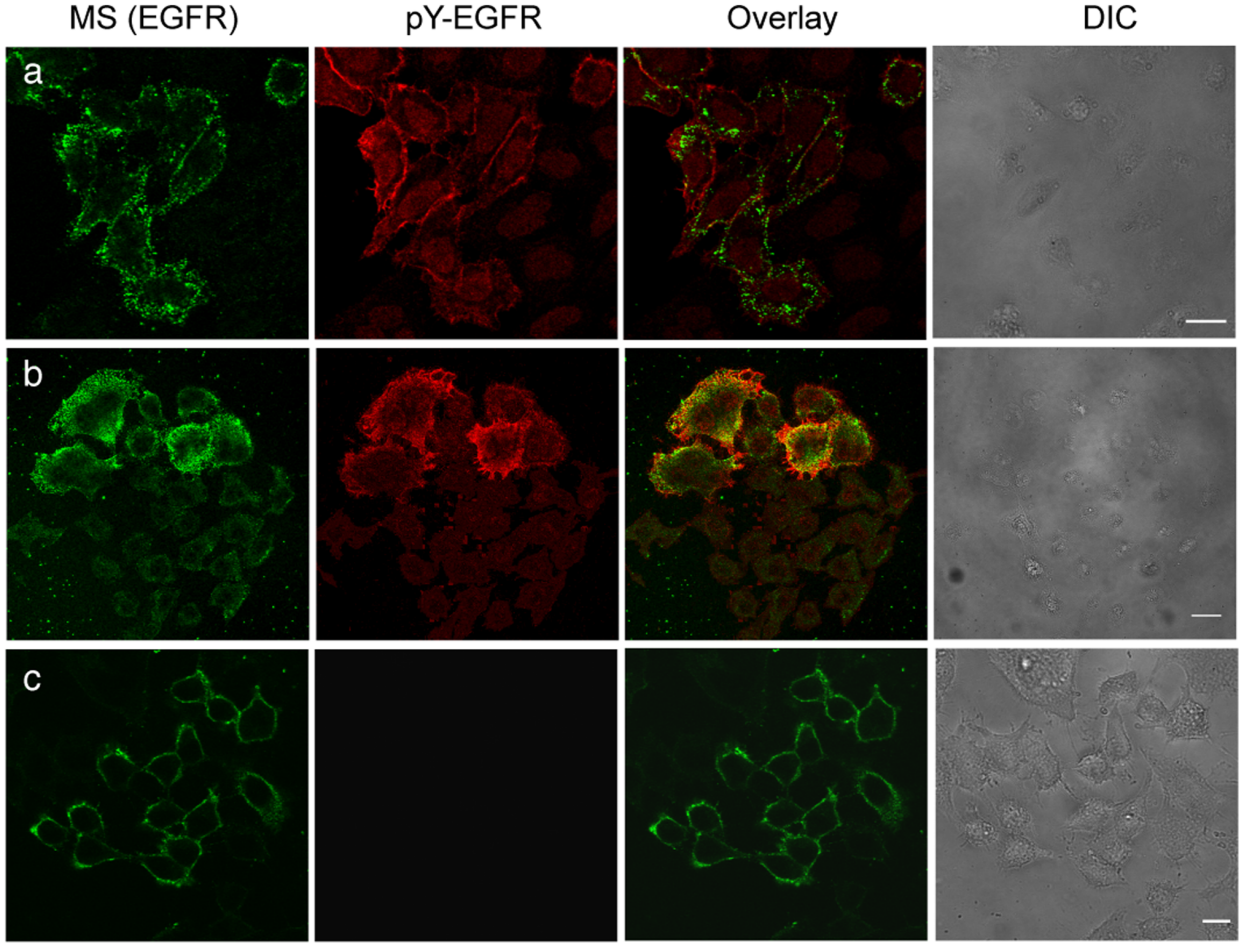
Magnetic activation is not dependent upon the targeting moiety. Stably transfected HeLa cells expressing chimeric ACP-EGFR, enzymatically modified by biotin CoA and carrying bound Strv-SPIONs. (a) 30 seconds, (b) 180 seconds of magnetization, (c) no magnetization. Green channel, Strv-SPION-biocytin-Alexa546; red channel, phosphorylated-EGFR (pY-EGFR 1068 and GARIG-CY5), overlay of green and red channels. Scale bar, 10 µm.

A similar clustering and activation of the EGFR was induced by strv-SPIONs targeted to the chimeric EGFR with the biotinylated ACP tag (Figure 6). We conclude that higher-order clustering of the receptor is sufficient for activation, i.e. does not require a unique orientation of the extracellular domain or binding of a large antibody molecule.

## Discussion

Localized activation of EGFR induced by EGF-coated magnetic microspheres (∼1 µm) has been shown to occur exclusively at the attachment loci on the cell membrane [42], [43], [44]. In the current study, we explored the applicability of much smaller superparamagnetic NPs lacking a natural ligand for the receptor and targeted to the EGFR with a monoclonal antibody that actually blocks ligand binding. The aim was to assess whether local clustering of the receptor by itself leads to activation and downstream signaling.

We demonstrated that simple bivalent coupling by streptavidin of the biotinylated antibody or targeting SPIONs to the cell membrane did not result in EGFR activation. However, exposure of the targeted SPIONs to a strong magnetic field resulted in a time-dependent and ligand-independent EGFR activation. MS consist of individual magnetic domains; a strong magnetic dipolar interaction is induced by the external magnetic field, which propagates and causes clustering of closely placed particles [45]. Exposure of cells to a magnetic field in the presence of non-targeted SPIONs failed to activate the EGFR.

Cells respond to mechanical stimuli through integrin signaling involving focal adhesion kinases (FAK) [46]. However, the forces required for conventional cellular mechanotransduction through integrins are on the order of pN to nN [46], [47], [48]. Surprisingly, the magnitude of forces measured in this study for activation of EGFR was estimated to be in the sub-pN range, (Supporting Information S1), i.e. considerably less than the force required for integrin mediated mechanotransduction.

Recently Rauch et al. [49] assayed the ability of dextran-coated SPIONs with various surface charges to activate ERK, AKT and EGFR in breast and colon cancer cells either expressing a metastatic RAS mutation or without this mutation. They found strong activation of the RAS pathway in the mutated cells in the absence of a magnetic field. In the absence of a magnetic field the epidermoid carcinoma cell line A431 did not show uptake or activation of our targeted SPIONs after incubations of 15 min at 37°C (Figure S5.) However, a slow uptake of the SPIONs, presumably by macropinocytosis, occurred upon prolonged incubation. We did not investigate cells with RAS mutations and thus cannot comment about whether non-dextran coated SPIONs might also effect a similar response as that seen by Rauch *et al* in such cells. The surface charge and structure of NPs are important determinants for their interaction with cells. This issue constitutes an important area of investigation.

Another publication featured gold-coated SPIONs coated with a saturating concentration of C225 monoclonal anti-EGFR SPIONs to study the downregulation of the EGFR after prolonged incubation (72 hours) in the absence of an external field whereby the cells autophagosized the particles [50]. C225 was originally characterized for its ability to block ligand binding to the EGFR, to arrest cell proliferation and to promote tumor killing [28], [37]. Coupling of the antibody to the NPs resulted in a two-fold increase in cell killing.

The absolute magnetization of MS depends on the magnetization distance and the strength of the applied magnetic field. We achieved full activation of the EGFR on A431 cells with an application of the magnetic field for 3 min at the employed field strength. The same downstream signaling cascade was promoted by magnetic activation with the SPIONs both using antibody targeting or direct binding of streptavidin SPIONs to the biotinylated chimeric EGFR.

The requirement for receptor-receptor interaction in the activation of signal transduction is a signature event of many receptor tyrosine kinases [52] and T cell receptors [53]. Cells with both high and low expression levels of EGFR (A431cells with 2·10^6^ compared to HeLa cells with 5·10^4^) were activated by SPIONs, leading to the conclusion that high *local* density (in the absence of the natural ligand) suffices for activation of the EGFR and its downstream signaling cascades. Thus, the data reinforce the hypothesis that upregulation of EGFR, interactions with other cell membrane components or sequestration in particular membrane microdomains leading to functional receptor clusters can potentiate cellular growth and migration of cells.

### Conclusions

We have demonstrated that physiologically important signaling receptors can be mechanically activated using magnetic nanotechnology. SPION technology constitutes a valuable tool for manipulating cellular processes in order to study and/or modulate functional states. In some human cancers receptor tyrosine kinases such as EGFR are in a constitutively active form due to mutations that abrogate the quiescent, non-aggregated state of the receptor. Our results provide direct evidence for a possible additional mechanism, i.e clustering per se, for the ligand free activation of EGFR in cancer cells expressing high levels of the wild-type receptor. Specific targeting of magnetized superparamagnetic NPs to the EGFR actuates the normal signaling cascade via the induction of a mechanical force of very low magnitude (sub-pN). It follows that magnetic switches used in conjunction with an external magnetic field may be used to control cellular functions *in vivo*. In particular, it may be possible to specify the location and strength of a signaling mechanism with magnetic control devices or to specifically deliver SPIONs into cells by such techniques. EGFR and other ErbB family members are upregulated in approximately 50% of all human tumors [51]. A prominent example is glioblastoma. Kantelhardt *et al*. [54] demonstrated that glioma cells expressing highly upregulated EGFR, in contrast to normal brain tissue lacking this receptor, were selectively labeled by quantum dots specifically targeted to the EGFR. SPIONs can be coupled to chemotherapeutic drugs, and animal studies show that uptake into tumors can be promoted by focused magnetic fields [55]. Thus, MS of the type described in this report may constitute useful vehicles for specifically targeting chemotherapeutic drugs to residual tumor cells after surgical resection.

## Materials and Methods

### Synthesis of Superparamagnetic Iron Oxide Nanoparticles and Surface Modification with Streptavidin

Superparamagnetic iron oxide NPs (SPIONs) were synthesized by alkaline coprecipitation of magnetite [56] from a mixture of Fe^3+^ and Fe^2+^ salts (FeCl_3_ and FeCl_2_ in 2∶1 molar ratio) followed by addition of concentrated NH_4_OH solution (25% w/v). The size of fractionated SPIONs was monitored by the transmission electron microscope (Jeol) operated at 120 kV and by dynamic light scattering (Malvern instruments) (Figure S1 a–d and e). SPIONS of 10–15 nm diameter were covalently coupled to streptavidin such that most particles had one or fewer streptavidin molecules (see Supporting Information S1 for details). Purified strv-SPIONs were resuspended in 20 mM Na-PO_4_, pH 7.6, buffer and further used for synthesis of the magnetic switches (MS).

### Preparation of Magnetic Switches

Magnetic switches (MS) capable of targeting EGFR were prepared (except when cells expressed ACP-EGFR, see below) by incubating a 5-fold molar excess of strv-SPIONs, in terms of biotin binding capacity, with biotinylated anti-EGFR monoclonal antibody 528 (Dianova) in 20 mM Na-PO_4_ buffer (pH 7.4) at 25°C for 15 min. Finally, the MS were incubated with a two-fold molar excess of biocytin-Alexa fluorophore (Invitrogen) (in terms of biotin binding capacity of strv-SPION) for 15 min and washed by magnetic separation several times to remove unbound fluorophore.

### Covalent Labeling of Chimeric ACP-EGFR with Biotin-CoA on Expressing Cells

Biotin-CoA was synthesized and purified by HPLC C_18_ chromatography as described in the literature [57]. We constructed a chimeric ErbB1 with an acyl carrier protein tag [58] between the signal peptide and the ErbB1 mature protein sequence. The serine that accepts a CoA derivative is located at position 36 in the sequence of the mature expressed protein. The 78 amino acid DNA sequence for the ACP tag was extracted from the AGA- plasmid (kind gift of N. Johnsson) by PCR and inserted between the signal sequence and the amino terminus by creating a new Nhe1 restriction enzyme cleavage site using site-directed mutagenesis of wild-type ErbB1 in pcDNA3 or a modified pcDNA3.1zeo^−^ plasmid. Stably-transfected Hela cell lines were selected by antibiotic resistance and single cell cloning. The chimeric EGFR behaves as wildtype EGFR as reported elsewhere [59].

Cells expressing the chimeric ACP-EGFR were labeled covalently with 1 µM CoA-biotin by enzymatic reaction at room temperature for 20 min with *E. coli* PPTase (phosphopantetheinyl transferase) [57], [60] which links biotin through a phosphodiester bond to the serine hydroxyl in the ACP tag. For experiments described in the text strv-SPION (10 µg/ml) NPs were added to the cells at 15°C for 15 min and excess SPIONs removed by washing before exposure of the cells to a magnetic field (Figure 6).

### Cell Culture

The following cell lines were used: A431, Human epidermoid carcinoma (ATCC CRL 1555) Clone E3 from E. Helmreich, Würzburg**;** Hela SS6; Hela stably expressing actin-GFP; and Hela stably expressing EGFR with an amino terminal ACP tag that can be covalently labeled by phosphopantetheinyl transferase with CoA–biotin. Cells were seeded on 24 mm square coverslips in complete DMEM and grown to 70% confluency. Cells were starved overnight and washed 3 times at 1 hr intervals prior to use in order to achieve a quiescent state and thus minimize the serum- or autocrine-induced activation of EGFR.

### Binding to Cells and Magnetization of MS

Cells were incubated in Tyrode’s buffer containing 0.1% BSA and 20 mM glucose at 15°C for 15 min with MS (5 µg/ml ≈ 6 nM Mab) and excess, unbound MS were removed by 3 washes with the same buffer. MS were magnetized by placing 4 permanent Neodymium magnets in a quadrapole configuration on the 4 sides of the glass coverslip at room temperture. After the allotted magnetization time coverslips were removed from the magnetic field and immediately fixed with 3.7% paraformaldehyde for 30 min on ice (unless otherwise indicated in the text) and then stained for activated EGFR or other cellular proteins as indicated in the figure legends.

### Detection of Phosphorylation of EGFR (pY-EGFR) by Immunofluorescence

Cells were fixed in 3.7% PFA for 30 min at 4°C, permeabilized and blocked with 0.1% Tween 20, 1% BSA in PBS at room temperature for 1 hr followed by incubation with rabbit anti-phosphotyrosine EGFR (pY-EGFR 1068 or pY-EGFR 1148 (Cell Signaling Technology) at 100–300 ng/ml. Primary antibodies were detected using secondary F(ab)_2_ fragments of goat anti-rabbit IgG (GARIG) labeled with Cy3 or Cy5 (Jackson Labs) at 1 µg/ml. Coverslips were mounted on microscope slides in Mowiol (Polysciences).

### Western Blot Analysis

For western blot analysis, A431 cells were grown in 24 well plates and treated with MS in the presence or absence of a magnetic field. Total cell lysates were obtained with Phosphosafe Reagent (Novagen) and equal amounts of protein were separated by a polyacrylamide gel electrophoresis, blotted to PVDF membranes and probed with rabbit primary antibody (pY-EGFR 1148, Cell Signaling Technology) and HRP-conjugated goat anti-rabbit IgG. Signal was developed by chemiluminescent (Supersignal West Pico, Pierce) and imaged with Kodak Biomax Xray film.

### Detection of MS in Early Endosomes

Magnetized cells were fixed after an additional incubation for 20 min at 37°C after removal from the magnetic field. Early endosomes were visualized by indirect immunofluorescence using 250 ng/ml rabbit polyclonal antibody (Abcam) against early endosomal antigen (EEA1) and goat anti-rabbit-Cy3 antibody (Jackson Labs).

### Shc Activation and Recruitment Assay

After binding of MS and magnetization, A431 cells were incubated for 10 min at 37 °C before fixation. Cells were stained with rabbit anti-phosphotyrosine 317 Shc antibody (Cell Signaling Technology) and GARIG-Cy5 (Jackson Labs). The distributions of Alexa 488 MS signal and Shc signal were detected by GARIG-Cy5. In the parallel control experiment the same incubation conditions were used without the application of a magnetic field.

### Confocal Microscopy

Confocal laser scanning fluorescence microscopy was carried out with an LSM 510 Meta system (Carl Zeiss, Jena) using a 63′, 1.4 NA Plan-Apochromat oil immersion objective. MS (Alexa 488) were excited at 488 nm with the Argon ion laser and detected with a 520/30 nm bandpass filter. GARIG-CY3 was excited with a 532 nm DSSP laser and its emission detected >585 nm. GARIG-Cy5 was excited with a 633 nm HeNe laser and its emission detected >650 nm. Images were recorded with 4 fold averaging in the XY dimension with 2 s sampling time per frame. All fluorescence images were recorded in multichannel mode in order to avoid the crosstalk between channels. Transmission images were acquired in DIC mode with either the 488 nm or the 532 nm laser line.

### Image Processing

Antibody and MS fluorescence signals were quantified using thresholding to segment images after background subtraction using the public domain NIH image J programs (available on the Internet at http://rsb.info.nih.gov/nih-image). Colocalization analyses of confocal stacks of 0.3 µ-separated images were performed after deconvolution with Huygens image processing software (Scientific Volume Imaging, Netherlands) and two-dimensional histograms representing the distributions plotted from these data. Mander’s coefficients were determined from deconvolved stacks by an Image J plugin.

### Scanning Electron Microscopy

SEM imaging of MS targeted to EGFR on A431 cell surfaces was carried out with a Hitachi S 5500 system operated at 30 kV. Cells were grown on 1 cm^2^ Si wafers treated with collagen, incubated with MS in the presence or absence of an external magnetic field and fixed with 2.5% EM grade glutaraldehyde followed by dehydration steps through a 30–100% ethanol gradient. In view of the low electron density and insulating nature of iron oxide, cells were treated with tannic acid and uranyl acetate for conductivity and contrast enhancement. Images were acquired over various magnifications in scanning mode with a field emission electron gun.

## Supporting Information

Figure S1 **TEM images of as synthesized SPIONs before (a,c) and after (b,d) magnetic fractionation.** SPIONs with fairly uniform size and shape were obtained after magnetic fractionation. (**e**) hydrodynamic size distribution from dynamic light scattering of magnetically fractionated SPIONs with the mean diameter of ∼ 15 nm.
(TIF)

Figure S2 **Targeted MS binding to A431 cells. (a)** Single confocal section near the cell attachment surface showing the fluorescence signal from Mab528-biocytin488 labeled MS from a confocal Z-stack. **(b)** DRAQ5 DNA fluorescence staining. **(c)** Overlay image from (a) and (b). **(d)** DIC image for (a and b). Scale bar 20 m. **(e)** Image of cells incubated with Alexa488-biocytin Strv-SPION lacking the targeting by anti-EGFR MAb, upper panel, 488 image, **(f)** DIC of cells in e. Similar sensitivity for the imaging of Alexa 488 fluorescence channel was used in a and e. Scale bar, 10 µm.
(TIF)

Figure S3 **Magnetization curve of SPIONs measured at room temperature (open circles).** Solid line, fit to the Langevin equation weighted by a lognormal size distribution.
(TIF)

Figure S4 **(a)** Distribution of the magnetic diameter of the population of SPIONs calculated based on magnetic properties. **(b)** Dependence of dipolar interaction force between neighboring SPIONs as a function of separation distance.
(TIF)

Figure S5 **Magnetic switches induce receptor activation only after exposure to a magnetic field.** A431 cells bound by MS and incubated for 15 min at 37°C either after exposure to a magnetic field for 30 s (panels **a** and **b**) or without exposure to a magnetic field (panels **c** and **d**). Galleries show every second confocal section of an image stack of 34 sections, each subimage is 71 µm square, 1 µm = 7.17 pixels. **a** and **c**, fluorescence of MS (stAv-SPIONs coupled with anti-EGFR 528 and loaded with 488 biocytin). **b** and **d**, immunofluorescence of pY-EGFR.
(TIF)

Figure S6 **MS cluster formation and dissociation dynamics.** Simulation of MS clusters formation and dissociation dynamics upon application and removal of a magnetic field (see text for the equations used).
(TIF)

Figure S7 **Lack of activation of EGFR induced by 20 min incubation after binding of streptavidin to cells saturated by biotinylated Mab 528.** Left, streptavidin signal; center, lack of signal from antibody for activated pY-EGFR; right, DIC image.
(TIF)

Figure S8 **Confocal immunofluorescence images of magnetic field induced activation of EGFR in HeLa cells.** (**a**) Green channel, MS bound to EGFR on cell membrane; (**b**) red channel, pY-EGFR; (**c**) overlay of green and red channels; (d) DIC image.
(TIF)

Supporting Information S1 **Magnetic characterization and calculations on magnetic dipolar forces.**
(PDF)

## References

[1] Alivisatos AP, Gu W, Larabell C (2005) Quantum Dots as cellular probes. Annu Rev Biomed Eng 7: 55–76. 16004566 10.1146/annurev.bioeng.7.060804.100432

[2] Kim J, Lee JE, Lee SH, Lee JH, Park TG, et al (2008) Designed fabrication of a multifunctional polymer nanomedical platform for simultaneous cancer- targeted imaging and magnetically guided drug delivery. Adv Mater 20: 478–483.

[3] Jun YW, Seo JW, Cheon J (2008) Nanoscaling Laws of magnetic nanoparticles and their applicabilities in biomedical sciences. Acc Chem Res 41: 179–189. 18281944 10.1021/ar700121f

[4] Parak WJ, Pellegrino T, Plank C (2005) Labelling of cells with Quantum Dots. Nanotechnology 16: R9–R25. 21727419 10.1088/0957-4484/16/2/R01

[5] Colombo M, Carregal-Romero S, Casula MF, Gutierrez L, Morales MP, et al (2012) Biological applications of magnetic nanoparticles. Chem Soc Rev 41: 4306–4334 10.1039/c2cs15337h. 22481569 10.1039/c2cs15337h

[6] Pankhurst QA, Connolly J, Jones SK, Dobson J (2003) Applications of magnetic nanoparticles in biomedicine. J Phy D: Appl Phys 36: R167–R181 10.1088/0022–3727/36/13/201.

[7] Pankhurst QA, Thanh NTK, Jones SK, Dobson J (2009) Progress in applications of magnetic nanoparticles in biomedicine. J Phys D: Appl Phys 42: 224001.

[8] Perez JM, Josephson L, O’Loughlin T, Hogemann D, Weissleder R (2002) Magnetic relaxation switches capable of sensing molecular interactions. Nat Biotechnol 20: 816–820. 12134166 10.1038/nbt720

[9] Huang H, Delikanli S, Zeng H, Ferkey DM, Pralle A (2010) Remote control of ion channels and neurons through magnetic-field heating of nanoparticles. Nat Nanotechnol 5: 602–606. 20581833 10.1038/nnano.2010.125

[10] Matthews BD, Thodeti CK, Tytell JD, Mammoto A, Overby DR, et al (2010) Ultra-rapid activation of TRPV4 ion channels by mechanical forces applied to cell surface beta1 integrins. Integr Biol (Camb) 2: 435–442. 20725677 10.1039/c0ib00034e PMC3147167

[11] Metzger H (1992) Transmembrane signaling: the joy of aggregation. Journal of Immunology 149: 1477–1487. 1324276

[12] Mannix RJ, Kumar S, Cassiola F, Montoya-Zavala M, Feinstein E, et al (2008) Nanomagnetic actuation of receptor-mediated signal transduction. Nat Nanotechnol 3: 36–40. 18654448 10.1038/nnano.2007.418

[13] Cho MH, Lee EJ, Son M, Lee JH, Yoo D, et al (2012) A magnetic switch for the control of cell death signalling in in vitro and in vivo systems. Nat Mater 11: 1038–1043 10.1038/nmat3430. 23042417 10.1038/nmat3430

[14] Etoc F, Lisse D, Bellaiche Y, Piehler J, Coppey M, et al (2013) Subcellular control of Rac-GTPase signalling by magnetogenetic manipulation inside living cells. Nat Nanotechnol 8: 193–198 10.1038/nnano.2013.23. 23455985 10.1038/nnano.2013.23

[15] Hoffmann C, Mazari E, Lallet S, Le Borgne R, Marchi V, et al (2013) Spatiotemporal control of microtubule nucleation and assembly using magnetic nanoparticles. Nat Nanotechnol 8: 199–205 10.1038/nnano.2012.246. 23334169 10.1038/nnano.2012.246

[16] Lemmon MA (2009) Ligand-induced ErbB receptor dimerization. Exp Cell Res 315: 638–648. 19038249 10.1016/j.yexcr.2008.10.024 PMC2667204

[17] Jura N, Zhang X, Endres NF, Seeliger MA, Schindler T, et al (2011) Catalytic control in the EGF receptor and its connection to general kinase regulatory mechanisms. Molecular Cell 42: 9–22. 21474065 10.1016/j.molcel.2011.03.004 PMC3175429

[18] Lu C, Mi LZ, Schurpf T, Walz T, Springer TA (2012) Mechanisms for kinase-mediated dimerization of the epidermal growth factor receptor. J Biol Chem 287: 38244–38253 10.1074/jbc.M112.414391. 22988250 10.1074/jbc.M112.414391 PMC3488093

[19] Oda K, Matsuoka Y, Funahashi A, Kitano H (2005) A Comprehensive pathway map of epidermal growth factor receptor signaling. Mol Syst Biol 1: 2005.0010. 10.1038/msb4100014 PMC1681468 16729045

[20] Schulze WX, Deng L, Mann M (2005) Phosphotyrosine interactome of the ErbB-receptor kinase family. Mol Syst Biol 1: 2005.0008. 10.1038/msb4100012 PMC1681463 16729043

[21] Avraham R, Yarden Y (2011) Feedback regulation of egfr signalling: decision making by early and delayed loops. Nat Rev Mol Cell Biol 12: 104–117. 21252999 10.1038/nrm3048

[22] Citri A, Yarden Y (2006) EGF-ErbB Signalling: Towards the systems level. Nat Rev Mol Cell Biol 7: 505–516. 16829981 10.1038/nrm1962

[23] Yu X, Miyamoto S, Mekada E (2000) Integrin alpha 2 beta 1-dependent EGF Receptor activation at cell-cell contact sites. J Cell Sci 113: 2139–2147. 10825287 10.1242/jcs.113.12.2139

[24] Takahashi M, Yokoe S, Asahi M, Lee SH, Li W, et al (2008) N-glycan of ErbB family plays a crucial role in dimer formation and tumor promotion. Biochim Biophys Acta 1780: 520–524. 18036567 10.1016/j.bbagen.2007.10.019

[25] Alexi X, Berditchevski F, Odintsova E (2011) The effect of Cell-ECM adhesion on signalling via the ErbB family of growth factor receptors. Biochem Soc Trans 39: 568–573 10.1042/BST0390568. 21428941 10.1042/BST0390568

[26] Lax I, Fischer R, Ng C, Segre J, Ullrich A, et al (1991) Noncontiguous regions in the extracellular domain of EGF receptor define ligand-binding specificity. Cell Regul 2: 337–345. 1716463 10.1091/mbc.2.5.337 PMC361798

[27] Li S, Schmitz KR, Jeffrey PD, Wiltzius JJ, Kussie P, et al (2005) Structural basis for inhibition of the epidermal growth factor receptor by cetuximab. Cancer Cell 7: 301–311. 15837620 10.1016/j.ccr.2005.03.003

[28] Sato JD, Kawamoto T, Le AD, Mendelsohn J, Polikoff J, et al (1983) Biological effects in vitro of monoclonal antibodies to human epidermal growth factor receptors. Molecular Biology and Medicine 1: 511–529. 6094961

[29] Astsaturov I, Cohen RB, Harari P (2007) EGFR-targeting monoclonal antibodies in head and neck cancer. Curr Cancer Drug Targets 7: 650–665. 18045070 10.2174/156800907782418365

[30] Ng K, Zhu AX (2008) Targeting the epidermal growth factor receptor in metastatic colorectal cancer. Crit Rev Oncol Hematol 65: 8–20. 18006328 10.1016/j.critrevonc.2007.09.006

[31] Russell JS, Colevas AD (2012) The use of epidermal growth factor receptor monoclonal antibodies in squamous cell carcinoma of the head and neck. Chemother Res Pract 2012: 761518 10.1155/2012/761518. 23150825 10.1155/2012/761518 PMC3488396

[32] Shih AJ, Telesco SE, Radhakrishnan R (2011) Analysis of somatic mutations in cancer: molecular mechanisms of activation in the ErbB family of receptor tyrosine kinases. Cancers (Basel) 3: 1195–1231. 21701703 10.3390/cancers3011195 PMC3119571

[33] Zeineldin R, Ning Y, Hudson LG (2010) the constitutive activity of epidermal growth factor receptor vIII leads to activation and differential trafficking of wild-type epidermal growth factor receptor and ErbB2. J Histochem Cytochem 58: 529–541. 20159766 10.1369/jhc.2010.955104 PMC2874185

[34] Roca AG, Costo R, Rebolledo AF, Veintemillas-Verdaguer S, Tartaj P, et al (2009) Progress in the preparation of magnetic nanoparticles for applications in biomedicine. J Phys D: Appl Phys 42: 224002.

[35] Guardia P, Perez N, Labarta A, Batlle X (2009) Controlled synthesis of iron oxide nanoparticles over a wide size range. Langmuir 26: 5843–5847 10.1021/la903767e. 10.1021/la903767e 20000725

[36] Gill GN, Kawamoto T, Cochet C, Le A, Sato JD, et al (1984) Monoclonal anti-epidermal growth factor receptor antibodies which are inhibitors of epidermal growth factor binding and antagonists of epidermal growth factor binding and antagonists of epidermal growth factor-stimulated tyrosine protein kinase activity. J Biol Chem 259: 7755–7760. 6330079

[37] Masui H, Kawamoto T, Sato JD, Wolf B, Sato G, et al (1984) Growth inhibition of human tumor cells in athymic mice by anti-epidermal growth factor receptor monoclonal antibodies. Cancer Res 44: 1002–1007. 6318979

[38] Sakaguchi K, Okabayashi Y, Kido Y, Kimura S, Matsumura Y, et al (1998) Shc phosphotyrosine-binding domain dominantly interacts with epidermal growth factor receptors and mediates Ras activation in intact cells. Mol Endocrinol 12: 536–543. 9544989 10.1210/mend.12.4.0094

[39] Authier F, Chauvet G (1999) In vitro endosome-lysosome transfer of dephosphorylated EGF receptor and Shc in rat liver. FEBS Lett 461: 25–31. 10561490 10.1016/s0014-5793(99)01413-1

[40] Goh LK, Huang F, Kim W, Gygi S, Sorkin A (2010) Multiple mechanisms collectively regulate clathrin-mediated endocytosis of the epidermal growth factor receptor. J Cell Biol 189: 871–883 10.1083/jcb.201001008. 20513767 10.1083/jcb.201001008 PMC2878939

[41] Sorkin A, Goh LK (2009) Endocytosis and intracellular trafficking of ErbBs. Exp Cell Res 315: 683–696. 19278030 10.1016/j.yexcr.2008.07.029 PMC2605728

[42] Lidke DS, Nagy P, Heintzmann R, Arndt-Jovin DJ, Post JN, et al (2004) Quantum Dot ligands provide new insights into ErbB/HER receptor-mediated signal transduction. Nat Biotechnol 22: 198–203. 14704683 10.1038/nbt929

[43] Brock R, Jovin TM (2001) Heterogeneity of signal transduction at the subcellular level: microsphere-based focal EGF Receptor activation and stimulation of Shc translocation. J Cell Sci 114: 2437–2447. 11559752 10.1242/jcs.114.13.2437

[44] Friedländer E, Nagy P, Arndt-Jovin DJ, Jovin TM, Szöllösi J, et al (2005) Signal transduction of ErbB receptors in Trastuzumab (Herceptin) sensitive and resistant cell lines: local stimulation using magnetic microspheres as assessed by quantitative digital microscopy. Cytometry 67A: 161–171. 10.1002/cyto.a.20173 16163699

[45] Lalatonne Y, Richardi J, Pileni MP (2004) Van der Waals versus dipolar forces controlling mesoscopic organizations of magnetic nanocrystals. Nat Mater 3: 121–125. 14730356 10.1038/nmat1054

[46] Overby DR, Matthews BD, Alsberg E, Ingber DE (2005) Novel dynamic rheological behavior of individual focal adhesions measured within single cells using electromagnetic pulling cytometry. Acta Biomater 1: 295–303. 16701808 10.1016/j.actbio.2005.02.003

[47] Wang N, Butler JP, Ingber DE (1993) Mechanotransduction across the cell surface and through the cytoskeleton. Science 260: 1124–1127. 7684161 10.1126/science.7684161

[48] Chen CS (2008) Mechanotransduction - a field pulling together? J Cell Sci 121: 3285–3292. 18843115 10.1242/jcs.023507

[49] Rauch J, Kolch W, Mahmoudi M (2012) Cell type-specific activation of AKT and ERK signaling pathways by small negatively-charged magnetic nanoparticles. Sci Rep 2: 868 10.1038/srep00868. 23162692 10.1038/srep00868 PMC3499776

[50] Yokoyama T, Tam J, Kuroda S, Scott AW, Aaron J, et al (2011) EGFR-targeted hybrid plasmonic magnetic nanoparticles synergistically induce autophagy and apoptosis in non-small cell lung cancer cells. PLoS One 6: e25507 10.1371/journal.pone.0025507. 22087216 10.1371/journal.pone.0025507 PMC3210119

[51] Hynes NE, Lane HA (2005) ErbB receptors and cancer: The complexity of targeted inhibitors. Nat Rev Cancer 5: 341–354. 15864276 10.1038/nrc1609

[52] Schlessinger J (2002) Ligand-induced, receptor-mediated dimerization and activation of EGF receptor. Cell 110: 669–672. 12297041 10.1016/s0092-8674(02)00966-2

[53] Balagopalan L, Barr VA, Samelson LE (2009) Endocytic events in TCR signaling: focus on adapters in microclusters. Immunol Rev 232: 84–98. 19909358 10.1111/j.1600-065X.2009.00840.x PMC3138075

[54] Kantelhardt SR, Caarls W, de Vries AHB, Hagen GM, Jovin TM, et al (2010) Specific visualization of glioma cells in living low-grade tumor tissue. PLoS One 5: e11323 10.1371/journal.pone.0011323. 20614029 10.1371/journal.pone.0011323 PMC2894859

[55] Seeney C, Ojwang JO, Weiss RD, Klostergaard J (2012) Magnetically vectored platforms for the targeted delivery of therapeutics to tumors: history and current status. Nanomedicine (Lond) 7: 289–299 10.2217/nnm.11.183 [doi]s. 22339137 10.2217/nnm.11.183

[56] Sahoo Y, Goodarzi A, Swihart MT, Ohulchanskyy TY, Kaur N, et al (2005) Aqueous ferrofluid of magnetite nanoparticles: Fluorescence labeling and magnetophoretic control. J Phys Chem B 109: 3879–3885. 16851439 10.1021/jp045402y

[57] Vivero-Pol L, George N, Krumm H, Johnsson K, Johnsson N (2005) Multicolor imaging of cell surface proteins. J Am Chem Soc 127: 12770–12771. 16159249 10.1021/ja0533850

[58] Duncan RR, Bergmann A, Cousin MA, Apps DK, Shipston MJ (2004) Multi-dimensional time-correlated single photon counting (TCSPC) fluorescence lifetime imaging microscopy (FLIM) to detect FRET in cells. J Microsc 215: 1–12 10.1111/j.0022–2720.2004.01343.x. 15230870 10.1111/j.0022-2720.2004.01343.x PMC1903372

[59] Ziomkiewicz I, Loman A, Klement R, Fritsch C, Klymchenko A, et al.. (2013) Dynamic conformational transitions of the EGF receptor (EGFR) in living mammalian cells determined by FRET and Fluorescence Lifetime Imaging Microscopy. Cytometry A doi: 10.1002/cyto.a.22311. 10.1002/cyto.a.22311 23839800

[60] Yin J, Straight PD, McLoughlin SM, Zhou Z, Lin AJ, et al (2005) Genetically encoded short peptide tag for versatile protein labeling by Sfp Phosphopantetheinyl Transferase. Proc Natl Acad Sci U S A 102: 15815–15820. 16236721 10.1073/pnas.0507705102 PMC1276090

