# Correction: Magnetic Nanoparticles as Mediators of Ligand-Free Activation of EGFR Signaling

**DOI:** 10.1371/annotation/a5aeb4a6-1ded-4dfd-9912-1aec923ca56e

**Published:** 2013-12-17

**Authors:** Atul A. Bharde, Raghavendra Palankar, Cornelia Fritsch, Arjen Klaver, Johannes S. Kanger, Thomas M. Jovin, Donna J. Arndt-Jovin

Figure S6 is a duplicate of Figure 5. Please see the correct Figure S6 here: 
