# Supplementary figures and images for: Magnetic Nanoparticles as Mediators of Ligand-Free Activation of EGFR Signaling
Source: PLoS One. 2013 Jul 23;8(7):e68879. doi: 10.1371/journal.pone.0068879 (PMC3720882; doi:10.1371/journal.pone.0068879)

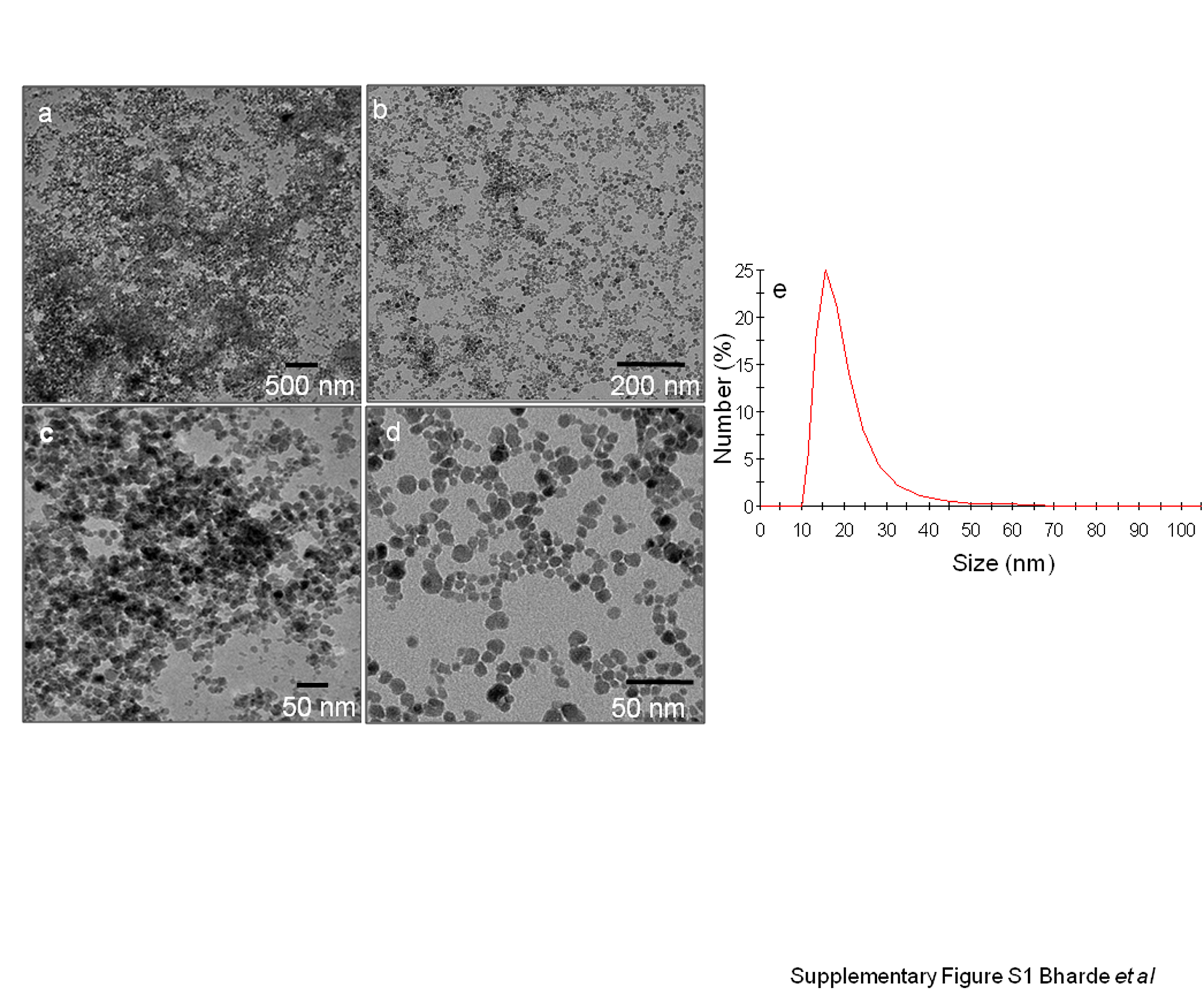

Supplement: Figure S1 — TEM images of as synthesized SPIONs before (a,c) and after (b,d) magnetic fractionation. SPIONs with fairly uniform size and shape were obtained after magnetic fractionation. (e) hydrodynamic size distribution from dynamic light scattering of magnetically fractionated SPIONs with the mean diameter of ∼ 15 nm. (TIF) [file pone.0068879.s001.tif]

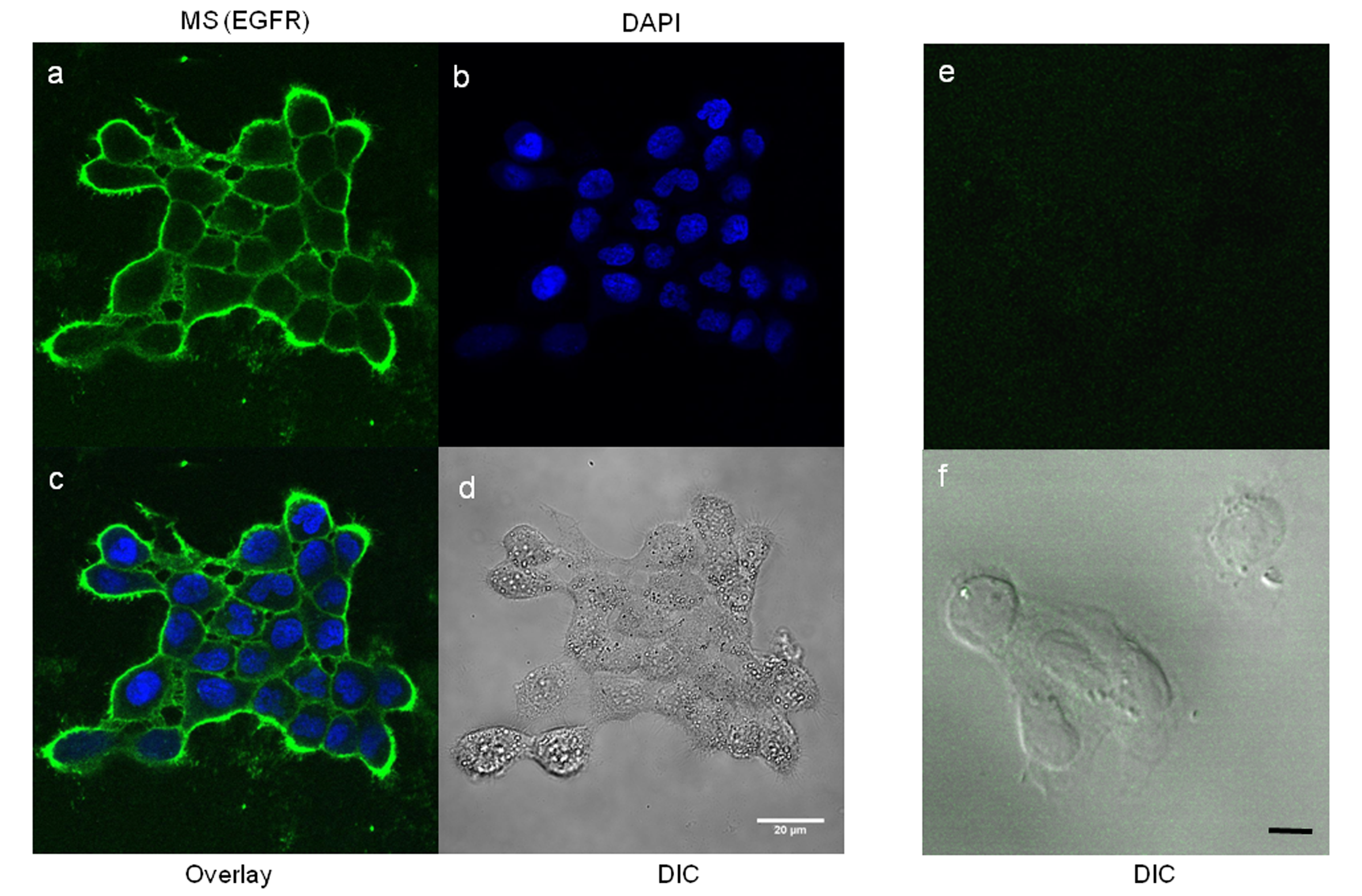

Supplement: Figure S2 — Targeted MS binding to A431 cells. (a) Single confocal section near the cell attachment surface showing the fluorescence signal from Mab528-biocytin488 labeled MS from a confocal Z-stack. (b) DRAQ5 DNA fluorescence staining. (c) Overlay image from (a) and (b). (d) DIC image for (a and b). Scale bar 20 m. (e) Image of cells incubated with Alexa488-biocytin Strv-SPION lacking the targeting by anti-EGFR MAb, upper panel, 488 image, (f) DIC of cells in e. Similar sensitivity for the imaging of Alexa 488 fluorescence channel was used in a and e. Scale bar, 10 µm. (TIF) [file pone.0068879.s002.tif]

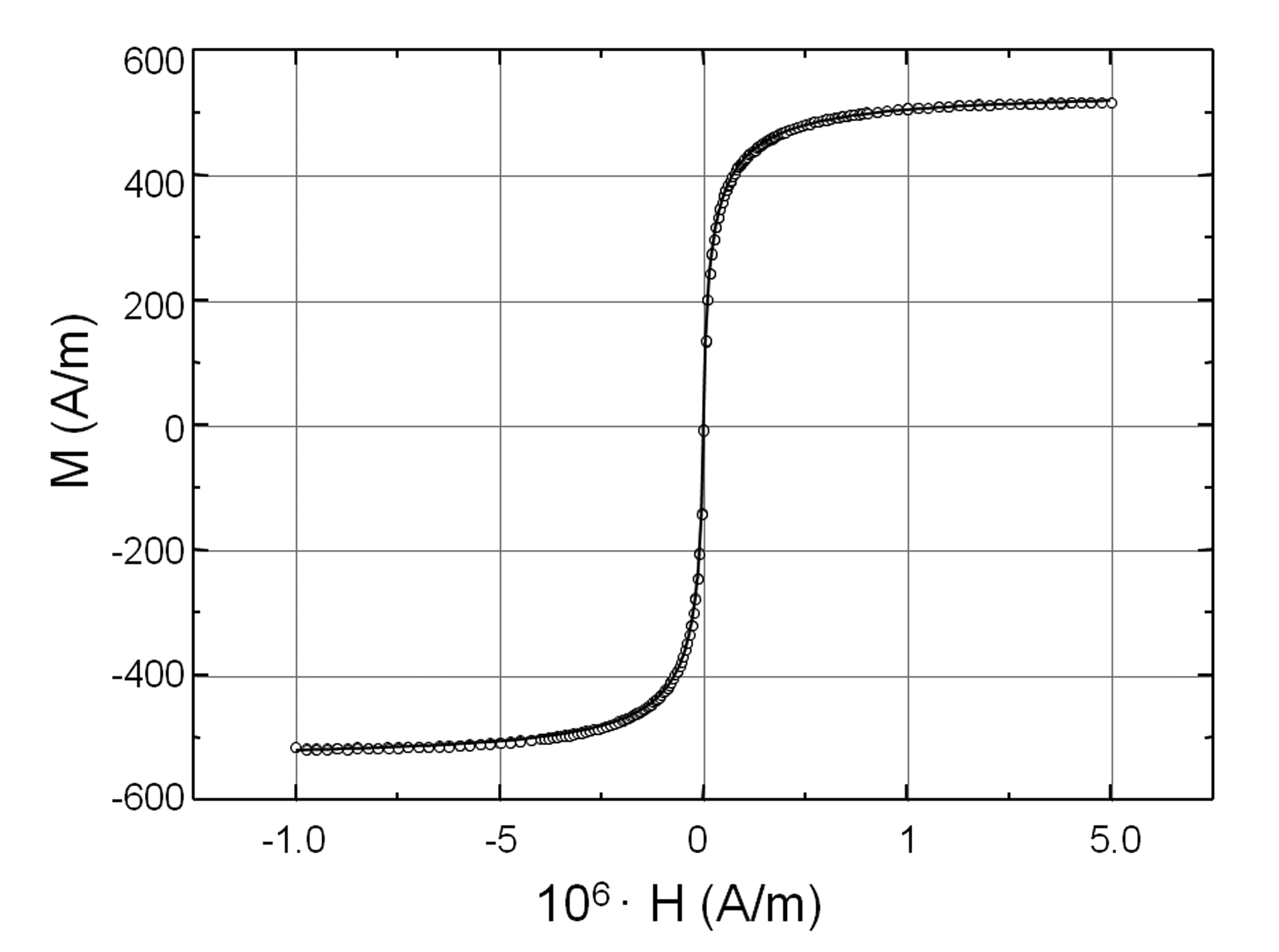

Supplement: Figure S3 — Magnetization curve of SPIONs measured at room temperature (open circles). Solid line, fit to the Langevin equation weighted by a lognormal size distribution. (TIF) [file pone.0068879.s003.tif]

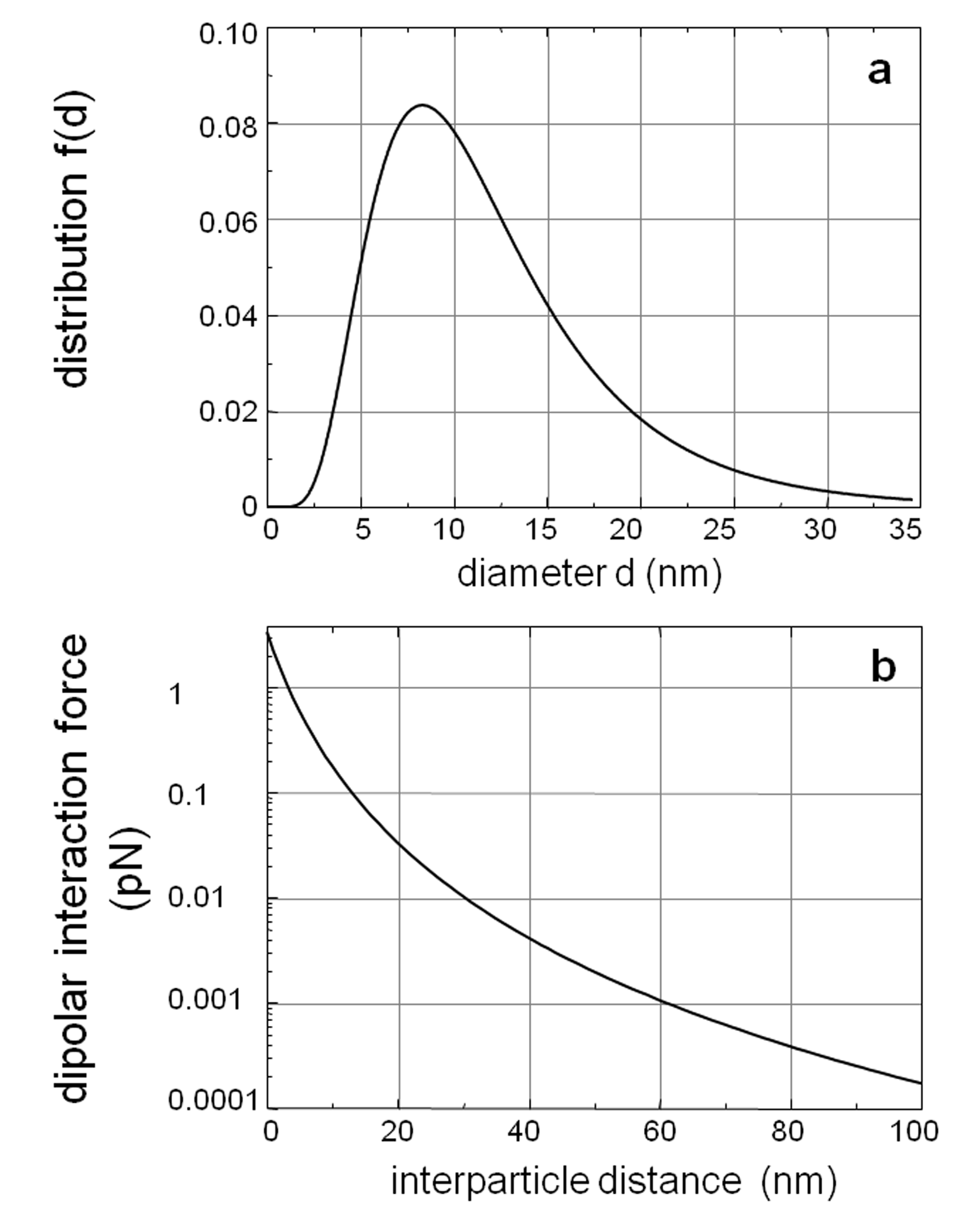

Supplement: Figure S4 — (a) Distribution of the magnetic diameter of the population of SPIONs calculated based on magnetic properties. (b) Dependence of dipolar interaction force between neighboring SPIONs as a function of separation distance. (TIF) [file pone.0068879.s004.tif]

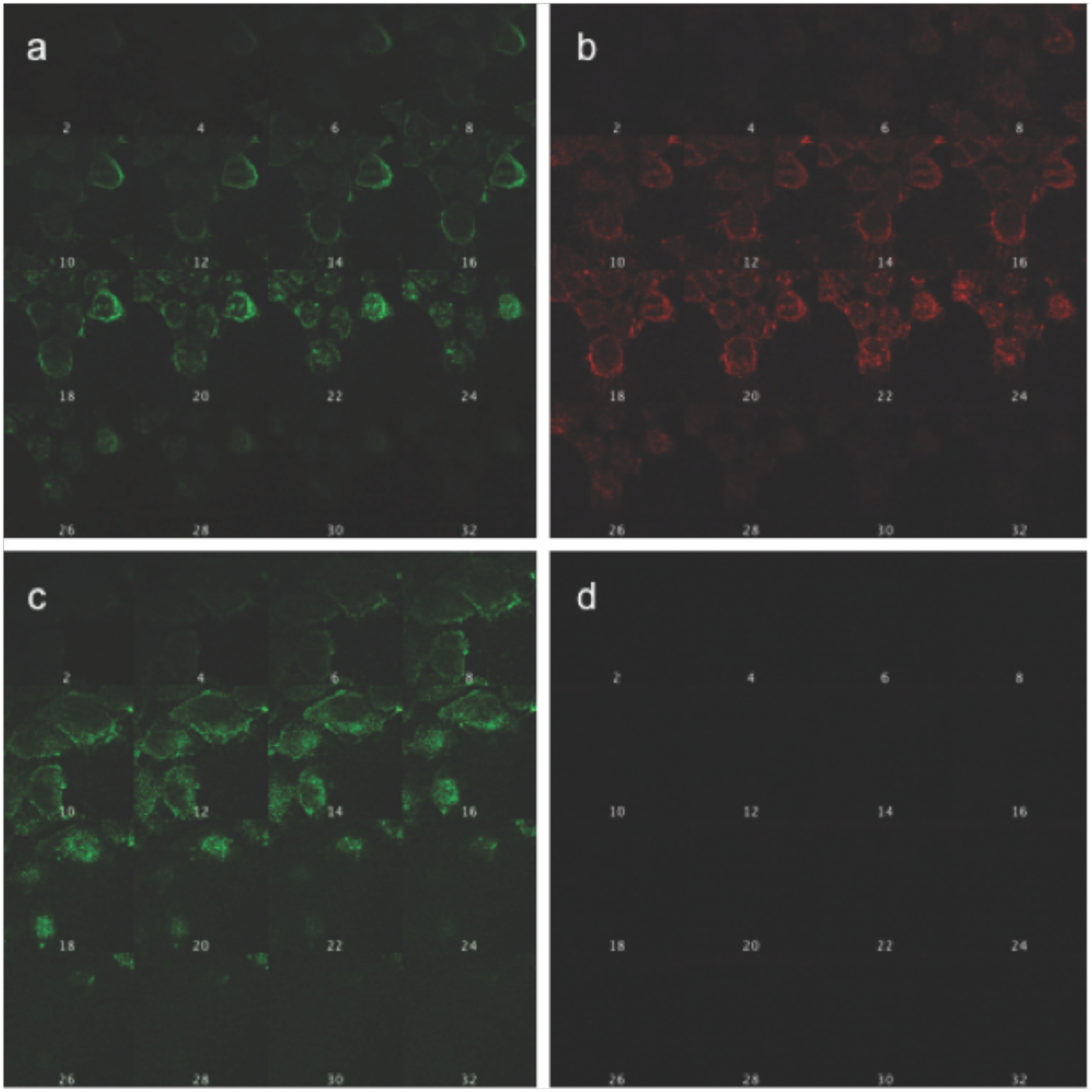

Supplement: Figure S5 — Magnetic switches induce receptor activation only after exposure to a magnetic field. A431 cells bound by MS and incubated for 15 min at 37°C either after exposure to a magnetic field for 30 s (panels a and b) or without exposure to a magnetic field (panels c and d). Galleries show every second confocal section of an image stack of 34 sections, each subimage is 71 µm square, 1 µm = 7.17 pixels. a and c, fluorescence of MS (stAv-SPIONs coupled with anti-EGFR 528 and loaded with 488 biocytin). b and d, immunofluorescence of pY-EGFR. (TIF) [file pone.0068879.s005.tif]

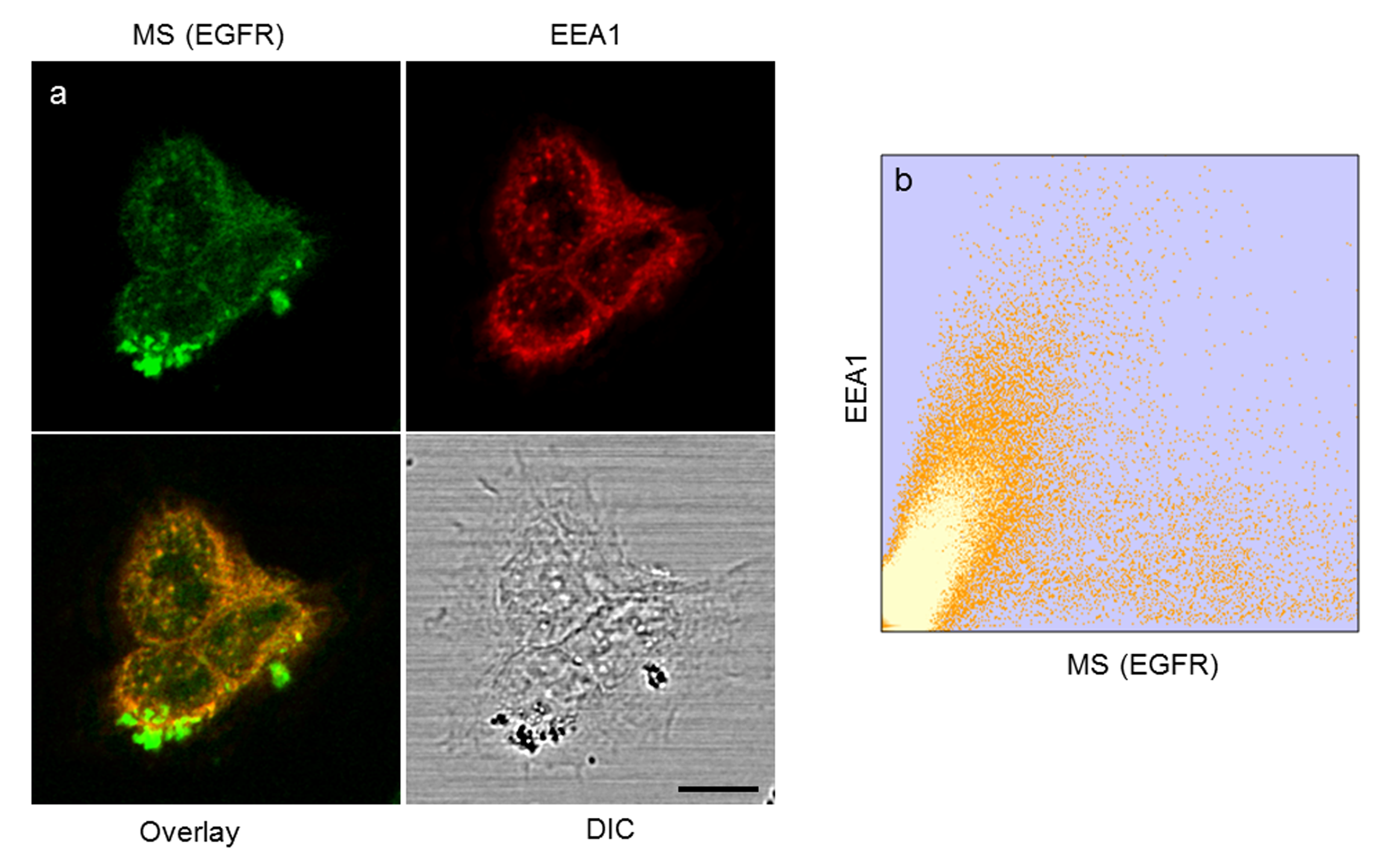

Supplement: Figure S6 — MS cluster formation and dissociation dynamics. Simulation of MS clusters formation and dissociation dynamics upon application and removal of a magnetic field (see text for the equations used). (TIF) [file pone.0068879.s006.tif]

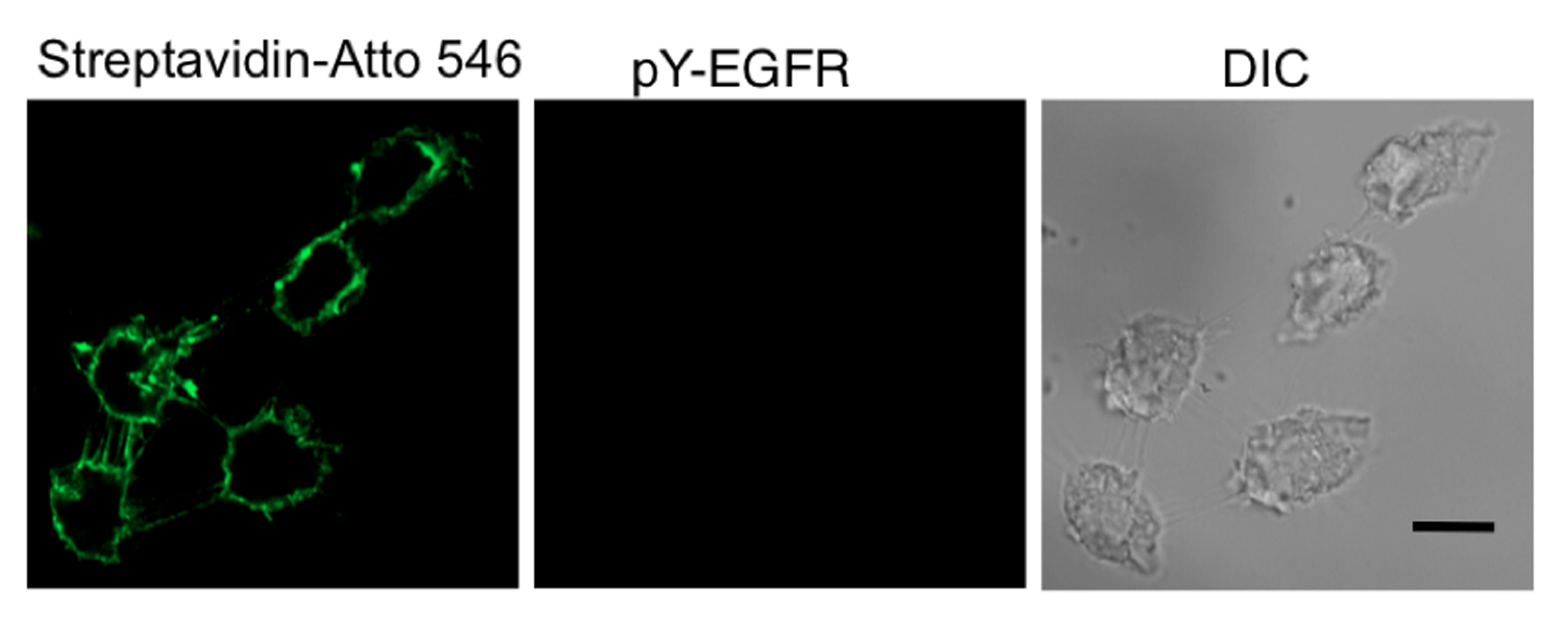

Supplement: Figure S7 — Lack of activation of EGFR induced by 20 min incubation after binding of streptavidin to cells saturated by biotinylated Mab 528. Left, streptavidin signal; center, lack of signal from antibody for activated pY-EGFR; right, DIC image. (TIF) [file pone.0068879.s007.tif]

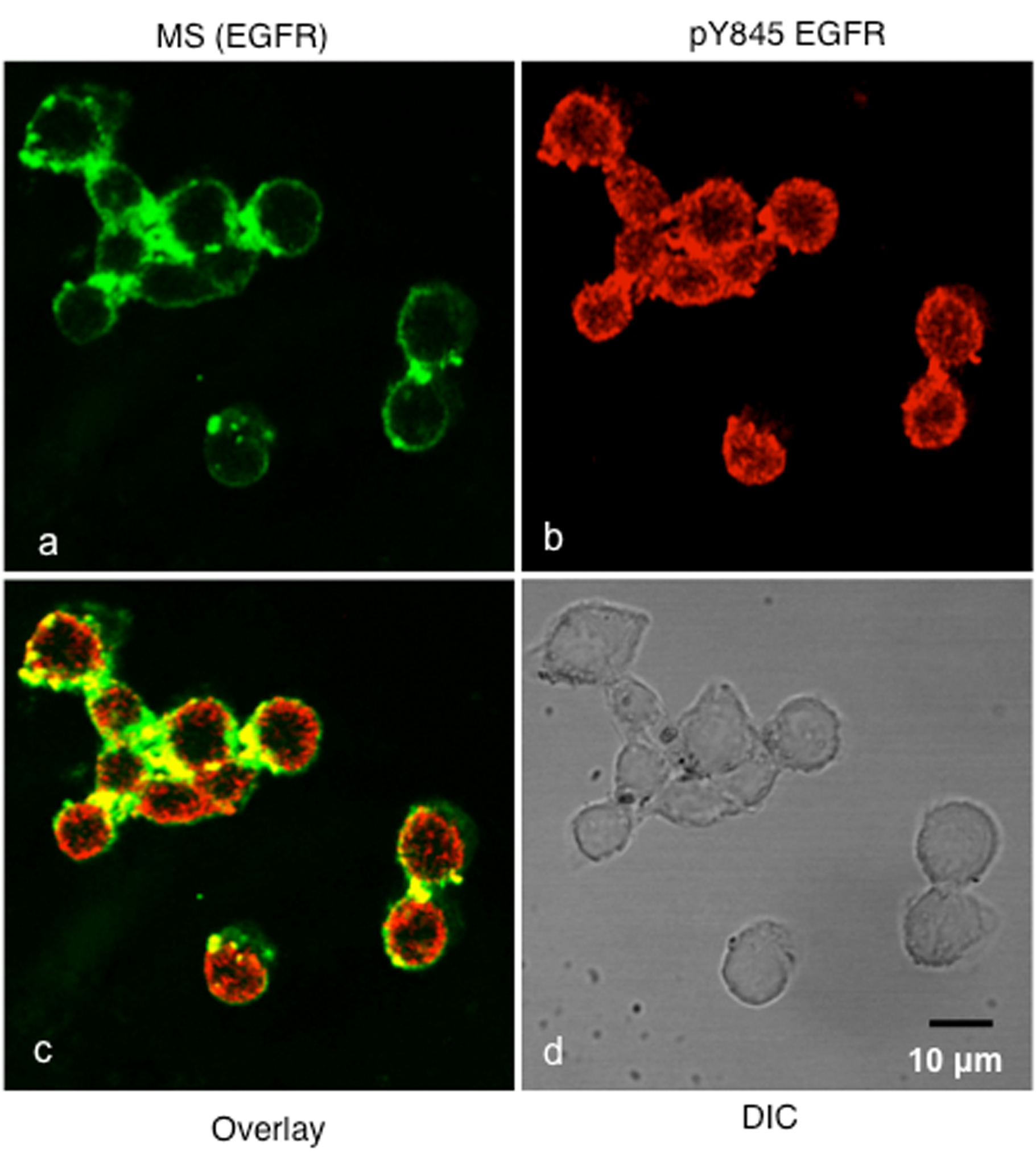

Supplement: Figure S8 — Confocal immunofluorescence images of magnetic field induced activation of EGFR in HeLa cells. (a) Green channel, MS bound to EGFR on cell membrane; (b) red channel, pY-EGFR; (c) overlay of green and red channels; (d) DIC image. (TIF) [file pone.0068879.s008.tif]
