# Supplementary material for: Magnetic Nanoparticles as Mediators of Ligand-Free Activation of EGFR Signaling
Source: PLoS One. 2013 Jul 23;8(7):e68879. doi: 10.1371/journal.pone.0068879 (PMC3720882; doi:10.1371/journal.pone.0068879)
Supplement: Supporting Information S1 — Magnetic characterization and calculations on magnetic dipolar forces. (PDF) [file pone.0068879.s009.pdf]

Supplementary Information

I. Synthesis of SPIONs and surface modification with streptavidin

In a typical reaction iron salts, 0.86g of $FeCl_2.4H_2O$ and $FeCl_3.6H_2O$ (1:2 molar ratio), were dissolved in 40 ml of deoxygenated deionized water in a three-necked-round bottom flask and heated at 80 ºC under argon atmosphere. While vigorously stirring, 5 ml of NH_4OH was introduced dropwise and heating was continued for 30 min to allow formation of nuclei. At this point, two ml of 1 M citric acid were introduced to the reaction mixture in order to stabilize the nanoparticles and the reaction mixture was heated to 90 ºC for another 120 min. Unreacted reagents were removed by dialysis against deionized water, after which the pH of the dispersion was ~ 7. TEM images of the synthesized SPIONs before size selection showed particles in the range of 4-20 nm diameter (Figure S1, a,b). The SPIONs were fractionated both by centrifugation and magnetic selection in 50 mM Na-PO_4 buffer (pH 7.6). The size distribution of the supernatant was monitored by DLS in a Malvern Zetasynthesizer. The fraction containing 10-20 nm SPIONs was selected for further experiments (Supplementary figure 1e). These particles were also measured by TEM (Supplementary Figure S1, c,d) showing sizes of 5-15 nm. The molar concentration of the particles was determined by the weight of 1 ml of SPIONs dispersed in pure water after drying.

Ten mg of SPIONs from a magnetically enriched fraction were suspended in 500 µl of 25 mM Na-PO_4 buffer (pH 7.6). Streptavidin was covalently coupled to carboxyl groups of SPIONs after activation by 10 mM EDC [1-ethyl-3-(3-dimethylaminopropyl) carbodiimide] and 5 mM N-hydroxysuccinimide (Thermo Scientific) for 30 min at 4° C. Streptavidin was added to the activated SPIONs to a final concentration of 0.5 µM (~ 30 µg /ml). This mixture was further incubated at 4° C for 6-12 h. Streptavidin coupled SPIONS (strv-SPIONs) were centrifuged at 12000 g for 20 min or magnetically concentrated and washed with 20 mM Na-PO_4 buffer of pH 7.6 to remove the excess unbound reagents.

II. Control Experiments

Dimerization of 528 MAb using streptavidin. A431 cells were incubated with biotinylated anti-EGFR MAb 528 (1 µg/ml) for 30 min at RT, washed and the Mab was crosslinked with

different concentrations of Atto565 streptavidin (1 ng/ml to 1 μg/ml) for 30 min, washed and assayed by immunofluorescence microscopy for specific phosphorylation of EGFR.

Incubation of cells with EGFR-bound targeted SPIONs at 37 ºC does not cause receptor activation. MS were bound to A431 cells for 15 min at 15 ºC, washed free of unbound MS and either incubated at 37 ºC without magnetization or after 30 s or 180 s with magnetization. Cells were fixed in 3.7% PFA, blocked and stained by indirect immunofluorescence for pY-EGFR as described in Materials and Methods. All images were recorded at the same sensitivity. Data for cells without magnetization and after 30 s magnetization are shown in Figure S5.

Non-specific binding of non-targeted SPIONs. The level of non-specific binding of magnetic nanoparticles was assessed by exposing the cells to Strv-SPIONs saturated with 488 biocytin under similar conditions as described in the Materials and Methods of the main text(NPs lacking the 528 MAb), followed by analysis of the 488 fluorescence signal by confocal microscopy under the same imaging conditions used with targeted MS (Figure S7).

III. Magnetic properties of SPIONs and calculations of dipolar magnetic forces

The magnetic properties of the synthesized magnetite particles were measured using a vibrating sample magnetometer (DMS 10 High Field VSM, ADE Technologies). The SPION solution (1.5 ml) was held in a glass sample holder. A diamagnetic correction was performed by subtracting the magnetization curve of an empty sample holder. Quasi-static magnetization curves were measured at room temperature (Supplementary Figure S3). The obtained magnetization curve did not show any hysteresis, consistent with superparamagnetic properties. Comparing the measured saturation magnetization of the sample $M_{SSample} = 0.52 \cdot 10^3$ A/m to the literature value[1] for bulk magnetite $M_{SBulk} = 4.8 \cdot 10^5$ A/m, a volume fraction of magnetite of 0.12 vol% was calculated. For monodisperse, non-interacting particles the magnetization curve can be analyzed using Langevin theory [2],

$$(1) \qquad M_{Sample} = M_{SSample}\left[\coth(\xi) - 1/\xi\right]$$

with $\xi = \frac{\mu_0 m H}{k_B T}$, M_{Sample}, the volume magnetization of the sample in A/m, $M_{SSample}$ the

saturation magnetization of the sample, μ_0 the vacuum permeability, m the magnetic dipole moment of a single particle (in units of Am^2), H the applied magnetic field in A/m and k_BT the thermal energy. The measured magnetization curve was fitted using the Langevin equation weighted by a lognormal size distribution of the magnetic diameters of the SPIONs (Figure S3). From the fit we obtained the distribution of the magnetic diameters of the SPIONs (Fig. S4a): 12 ± 7 nm.

In addition we obtained the magnetic dipole moment of the particles as $m = M_{SBulk}\pi d^3/6 = 4.5 \cdot 10^{-19}$ A m^2 (using an average particle diameter of 12.2 nm), a value close to reported literature values of $1.9 \cdot 10^{-19}$ A m^2 for a similar particle size [3]. Next, the magnetic dipole moments were used to estimate the magnetic dipole-dipole forces as well as the forces exerted on the particles due to the gradient in the external magnetic field.

In order to estimate the magnetic dipole-dipole forces that act between two MNP in the external magnetic field as well as the magnetic gradient force acting on a single MNP we performed finite element analysis of the experimental magnetic geometry. Calculations were carried out using Comsol Multiphysics (Comsol AB, Stockholm, Sweden) software. This calculation showed that the magnetic field, H, at the position of the cells (2 mm from the magnet) was $H = 3\times 10^5$ A/m. From Figure S1 it follows that at these field strengths the SPIONs were very close to saturation, implying that the amplitude of the magnetic dipole moments found above could be used to calculate the magnetic forces on the MNP. The magnetic dipole-dipole interaction force was calculated using the expression $F = (3/2)(\mu_0/\pi)m^2r^{-4}$ for the maximal force [3]. (The two dipole moments are aligned such that they point in the same direction and parallel to the line connecting the two dipoles.) At an average distance of $r \approx 40$ nm a magnetic force of $F \approx 10$ fN was calculated. The force exerted by the external magnet on an individual MNP was calculated according to

$$\vec{F} = \vec{\nabla}\left(\vec{m} \cdot \vec{B}\right) \quad (2)$$

From the finite element analysis, dB/dx = -87 T/m (at 2 mm from the magnet), from which, $F \approx 10$ aN, a negligible value compared to the dipole-dipole interaction force.

A criterion for magnetic field induced clustering is that the ratio λ between the magnetic interparticle interaction energy and the thermal energy exceeds unity [4]. This ratio is calculated as:

(3) $$\lambda = \frac{\mu_0}{2\pi} \frac{m^2}{r^3 k_B T}$$

where r is the particle-particle distance. In our case we found a maximum value of λ =6 for two SPIONs in contact, indicating that both the strength of the magnetic dipole moments and the external field were more than adequate to achieve stable clustering. The interaction energy between two particles is much lower than the thermal energy if the particles are separated by only a few nanometers (e.g. for a distance of 15 nm we find $\lambda = 0.5$) indicating that the long-range magnetic dipole-dipole interactions are negligible compared to the thermal energy. We conclude that the rate of clustering of the MS was determined by the Brownian motion.

IV. Simulations of the cluster formation dynamics and Brownian relaxation

2D Brownian dynamics simulations were performed using a locally generated software program to determine the degree of clustering of SPIONs in the presence and absent of a magnetic field. Briefly, SPION were modelled as magnetic dipoles with a given magnetic moment. Two types of inter-particle interactions were assumed for magnetic cluster formation simulations: magnetic dipole-dipole interactions [5], which are only present during the magnetic field, and repulsive excluded volume interactions. Simulations were then followed by cluster analysis to count the number of SPION that were associated with a cluster. A cluster was defined to consist of at least two SPION with a center-to-center distance smaller or equal to a single SPION diameter. The dipole-dipole interaction energy is given by the following equation [6]:

(4) $$U_{ij}^{dip} = \frac{\mu_0}{4\pi}\left(\frac{\vec{m}_i \cdot \vec{m}_j}{\left|\vec{r}_{ij}\right|^3} - \frac{3(\vec{m}_i \cdot \vec{r}_{ij})(\vec{m}_j \cdot \vec{r}_{ij})}{\left|\vec{r}_{ij}\right|^5} \right)$$

on the other hand, the repulsion is characterized by a Weeks-Chandler-Andersen potential [7] and given by the equation:

(5) $$U_{ij}^{Vol} = \begin{cases} 4\varepsilon\left[\left(\frac{\sigma}{\left|\vec{r}_{ij}\right|}\right)^{12} - \left(\frac{\sigma}{\left|\vec{r}_{ij}\right|}\right)^{6}\right] + \varepsilon & \text{if } \left|\vec{r}_{ij}\right| \le 2^{1/6}\sigma \\ 0 & \text{if } \left|\vec{r}_{ij}\right| > 2^{1/6}\sigma \end{cases}$$

For simulations, N particles were distributed randomly in a square area of length L. The particles were allowed to move in the 2D plane. Rotation of the particles was not considered for the simulations. The displacement of each particle was then calculated with the equation:

(6)
$$\Delta\vec{r}_i = \frac{\vec{F}_i^{dip} + \vec{F}_i^{vol} + \vec{F}_i^{rand}}{\gamma}\Delta t$$

where, γ is the friction constant of translational movement, F^{dip} the dipole-dipole interaction force, F^{vol}, the excluded volume force, and F^{rand} ^{i}is the Gaussian random force with mean zero and a standard deviation satisfying the fluctuation-dissipation relations. The temperature of the system was set at 300 K. For simulations we considered N = 600, L = 1 μm, σ = 8 nm, ε= 10^{-21} m^2s^{-1}, Δt = 10^{-7} s. The friction constant γ was calculated using $\gamma = k_BT/D$, with D the diffusion constant; we used D = 10^{-13} m^2s^{-1}, a value typical for the EGFR receptor in membranes [8]. All SPIONs were assumed to be saturated with a magnetization pointing in one direction in the plane. Each simulation consisted of 10^6 time steps covering 0.1 s. The trajectories of each SPION were stored for later analysis. After the simulations, we performed cluster analysis to count the number of SPIONs associated with a cluster.

At the beginning of the simulations, no magnetic field is present and SPIONs only exhibit Brownian motion. We considered two SPIONs to be clustered if their center-to-center distance was less than the diameter of a single SPION, such that some probability of transient clustering existed under the given experimental conditions. It should be noted that these clusters were not stable resulting in a "noisy" offset (Supplemenatary Figure S6). At t = 0 s the magnetic field was applied and all SPIONs were considered to be magnetized. As a result, SPIONs in close proximity were attracted to each other due to magnetic dipole-dipole interaction forces and formed stable clusters. The number of clustered SPIONs grew rapidly until the magnetic saturation where almost all the SPIONs were in clusters. From these calculations we obtained the characteristic times of ~ 10 ms for cluster formation.

The dynamics of cluster formation and cluster breakdown are determined by different processes. Both Neel and Brownian rotational relaxation result in a fast reorientation of the magnetic dipole moment of the MS. Upon application of the magnetic field, the dipole moments align themselves in the field direction, but assume a random orientation upon

removal of the field. The characteristic relaxation time for reorientation of the magnetic moment in superparamagnetic nanoparticles is a function of thermal energy, and is known as the Brownian relaxation time τ,

$$\tau = \frac{4\pi r^3 \eta}{k_B T} \quad (7)$$

where r is the hydrodynamic radius of the magnetic nanoparticle, η is the viscosity of the surrounding medium, k_B is Boltzmann's constant and T is temperature [9]. The calculated Brownian relaxation time for 10 nm particles was ~ 1 μs. Although Neel relaxation is usually faster or comparable to Brownian relaxation [3], the clustering dynamics in our experiments would have been mainly determined by the translational Brownian motion of the MS and the diffusion of the coupled EGFR in the cell membrane. Translational Brownian dynamics simulations confirmed that the translational relaxation would be much slower than rotational diffusion; i.e. clustered MS would require ~20 ms to dissipate and redistribute over the cell surface (Figure S6). A rapid randomization of the magnetic orientation of the MS was achieved within a short time after the removal of the magnetic field. However, as indicated in the main text, transphosphorylation (activation) of the receptor in the clusters occurred upon application of the magnetic field. Such receptors presumably continued signaling until being deactivated by cellular phosphatases acting at the plasma membrane or in internal vesicles.

Supplementary References

1. Goya GFB, TS; Fonseca, FC (2003) Static and ynamic magnetic properties of spherical magnetite nanoparticles. J Appl Phys 94: 3520-3528.
2. Langevin P (1905) Theory of Magnetism. J Physique 4: 678-693.
3. Chung SH, Hoffmann A, Bader SD, Liu C, Kay B, et al. (2004) Biological sensors based on Brownian Relaxation of magnetic nanoparticles. Appl Phy Lett 85: 2971-2973.
4. Dominguez-Garcia P, Melle S, Pastor JM, Rubio MA (2007) Scaling in the aggregation dynamics of a magnetorheological fluid. Phys Rev E Stat Nonlin Soft Matter Phys 76: 051403.
5. Cerda JJ, Kantorovich S, Holm C (2008) Aggregate formation in ferrofluid monolayers: simulations and theory. J Phys Condens Matter 20: 204125.
6. Jackson JD (1998) Classical electrodynamics: John Wiley & Sons, New York. 145-154 p.

7. Weeks JDC, D.; Andersen, HC (1971) Role of repulsive forces in determining the equilibrium structure of simple liquids. J Chem Phys 54: 5237-5247.
8. Lidke DS, Lidke KA, Rieger B, Jovin TM, Arndt-Jovin DJ (2005) Reaching out for signals: Filopodia sense EGF and respond by directed retrograde transport of activated receptors. J Cell Biol 170: 619-626.
9. Joonsik L, Yong-Ho C, Jongryoul K, Ki Hyeon K (2011) Comparison of the magnetic properties for the surface-modified magnetite nanoparticles. Magnetics, IEEE Transactions on 47: 2874-2877.
