# Supplementary figures and images for: Correction: Magnetic Nanoparticles as Mediators of Ligand-Free Activation of EGFR Signaling
Source: PLoS One. 2013 Dec 17;8(12):10.1371/annotation/a5aeb4a6-1ded-4dfd-9912-1aec923ca56e. doi: 10.1371/annotation/a5aeb4a6-1ded-4dfd-9912-1aec923ca56e (PMC3866305; doi:10.1371/annotation/a5aeb4a6-1ded-4dfd-9912-1aec923ca56e)

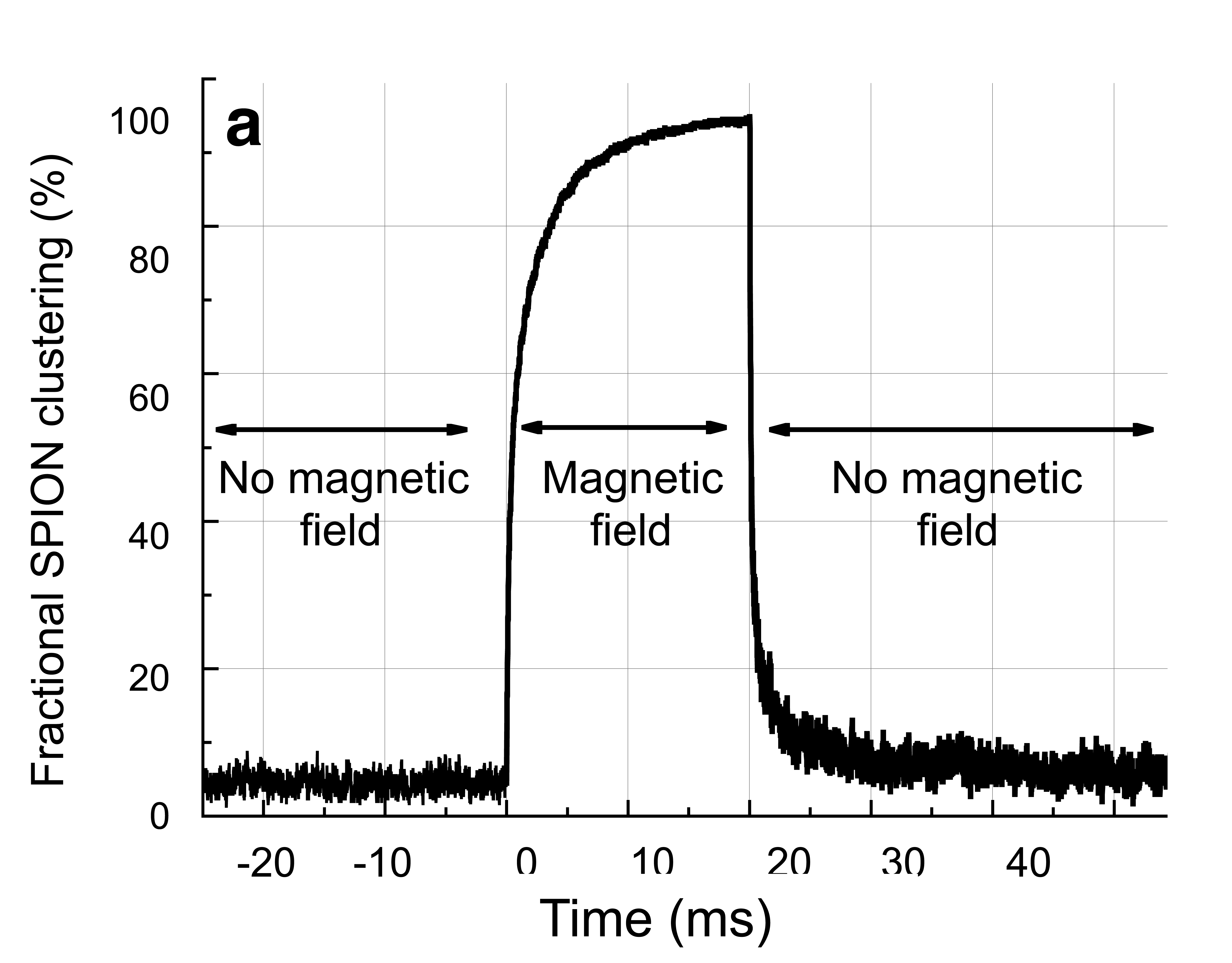

Supplement: Supplementary file 1 [file pone.a5aeb4a6-1ded-4dfd-9912-1aec923ca56e.s001.tif]
